# Motivational Salience Signal in the Basal Forebrain Is Coupled with Faster and More Precise Decision Speed

**DOI:** 10.1371/journal.pbio.1001811

**Published:** 2014-03-18

**Authors:** Irene Avila, Shih-Chieh Lin

**Affiliations:** Neural Circuits and Cognition Unit, Laboratory of Behavioral Neuroscience, National Institute on Aging, National Institutes of Health, Baltimore, Maryland, United States of America; University of Oregon, United States of America

## Abstract

The survival of animals depends critically on prioritizing responses to motivationally salient stimuli. While it is generally believed that motivational salience increases decision speed, the quantitative relationship between motivational salience and decision speed, measured by reaction time (RT), remains unclear. Here we show that the neural correlate of motivational salience in the basal forebrain (BF), defined independently of RT, is coupled with faster and also more precise decision speed. In rats performing a reward-biased simple RT task, motivational salience was encoded by BF bursting response that occurred before RT. We found that faster RTs were tightly coupled with stronger BF motivational salience signals. Furthermore, the fraction of RT variability reflecting the contribution of intrinsic noise in the decision-making process was actively suppressed in faster RT distributions with stronger BF motivational salience signals. Artificially augmenting the BF motivational salience signal via electrical stimulation led to faster and more precise RTs and supports a causal relationship. Together, these results not only describe for the first time, to our knowledge, the quantitative relationship between motivational salience and faster decision speed, they also reveal the quantitative coupling relationship between motivational salience and more precise RT. Our results further establish the existence of an early and previously unrecognized step in the decision-making process that determines both the RT speed and variability of the entire decision-making process and suggest that this novel decision step is dictated largely by the BF motivational salience signal. Finally, our study raises the hypothesis that the dysregulation of decision speed in conditions such as depression, schizophrenia, and cognitive aging may result from the functional impairment of the motivational salience signal encoded by the poorly understood noncholinergic BF neurons.

## Abstract 2

Avila and Lin report that the speed and variability of the decision-making process are tightly coupled and jointly determined by the motivational salience signal in the basal forebrain.

## Author Summary

Humans and animals face the constant challenge of identifying the subset of incoming sensory stimuli that are most behaviorally relevant and prioritizing behavioral responses accordingly. Critical to this decision is the ability to determine whether a stimulus is motivationally salient—that is, whether the stimulus predicts important behavioral outcomes such as reward or punishment. While it is generally assumed that motivational salience is related to faster decision speed and shorter reaction time, it remains unclear how motivational salience actually modulates the decision-making process. This study investigates how the motivational salience signal in the basal forebrain controls the fundamental properties of the decision-making process—decision speed and its variability. In rats performing a reward-biased simple reaction time task, we show that the basal forebrain motivational salience signal is associated with a faster and also precise decision speed. In support of a causal role for this association, artificially augmenting this basal forebrain motivational salience signal by electrical stimulation also leads to faster and more precise reaction times. These results suggest that decision speed and its variability are jointly determined by an early and previously unrecognized step in the decision-making process, dictated largely by the motivational salience signal encoded by poorly understood noncholinergic neurons in the basal forebrain.

## Introduction

The overall speed of information processing and decision-making has been studied for more than a century by measuring reaction time (RT) [1]–[3]. Significant increases in RT, reflecting a slower decision speed, represents a key feature in several conditions such as depression [4],[5], dementia [6],[7], schizophrenia [8]–[10], and cognitive aging [11],[12]. Therefore, it is important to understand how decision speed is modulated by cognitive variables and by underlying neural circuit mechanisms.

An important modulator of decision speed is motivational salience [13]–[16]. Determining whether a stimulus is motivationally salient—that is, whether the stimulus predicts important behavioral outcomes such as reward or punishment—allows humans and animals to select among incoming sensory information the subset of stimuli that are behaviorally relevant. Thus, motivational salience plays a key role in goal-directed decision-making to prioritize behavioral responses. As a result, neural correlates of motivational salience are commonly defined or inferred through the modulation of RT [13]–[15]. However, this logical interdependency poses a fundamental challenge in understanding the relationship between motivational salience and decision speed.

The alternative approach we took to avoid this circular logic was to investigate the relationship between RT and a neural correlate of motivational salience defined independently of RT. Recent studies identified a neural correlate of motivational salience in the basal forebrain (BF) [16], where a distinct group of BF neurons respond to motivationally salient stimuli that predict either reward or punishment with similar and robust phasic bursting responses [16]–[20]. The strength of the BF motivational salience signal, reflected by the amplitude of bursting response, is coupled with the overall response latency [16]. The same BF neurons also respond to primary reward and punishment with similar bursting response [16]. We hypothesize that the bursting response of BF neurons can translate the motivational salience signal into widespread modulation of cortical activity [21] and therefore represents an ideal candidate mechanism to increase decision speed and reduce RT. In support of this hypothesis, slowing of RT was observed following lesion [22]–[25] or inactivation [26],[27] of the BF.

Our approach to understand how BF motivational salience signal modulates decision speed was to first determine the part of RT variability that was correlated with, and likely modulated by, BF motivational salience signal while rats responded to two motivationally salient stimuli that predicted different amounts of reward. Second, we sought to determine the part of RT variability that was present in the face of constant BF bursting response, which does not reflect the moment-to-moment fluctuation of motivational salience and likely represents how RT is modulated by the intrinsic noise in the decision-making process. By partitioning RT variability into two distinct sources that were either correlated with or independent of BF motivational salience signal, we further investigated whether the strength of BF motivational salience signal modulated the magnitude of RT variability contributed by intrinsic noise. Finally, we tested whether the observed functional coupling between BF bursting response and RT represented a causal relationship using electrical microstimulation of the BF.

## Results

**Figure 1:**
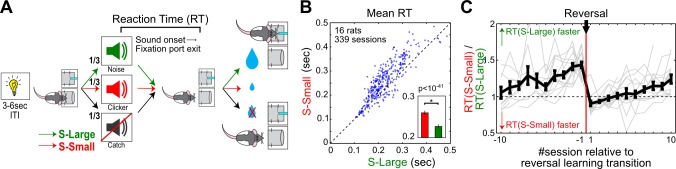
Reward-biased simple RT task. (A) Schematic of the reward-biased simple RT task. Rats initiated each trial by nosepoking in a fixation port following a trial start light signal. Inside the fixation port, three trial types—S-Large, S-Small, and Catch—were presented with equal probability and respectively associated with a large, small, or no reward in the adjacent port. RT was defined as the time between sound onset and fixation port exit. (B) Scatter plot of the mean RT in S-Large versus S-Small trials. Each dot represents one session from one rat (*n* = 339, 16 rats). Inset shows the overall mean ± sem (paired *t* test). (C) RT modulation as a ratio of mean RT between S-Large versus S-Small trials in each session around the reversal learning transition. Seventeen individual transition sequences (gray lines) with at least five sessions both before and after reversal learning were plotted, with the overall mean ± sem in black. In the first three sessions after the reversal learning transition, RT was faster toward S-Small, which predicted the larger reward before reversal. The RT difference grew larger with more training, and did not reach asymptotic level after 10 sessions.

To investigate the relationship between motivational salience and decision speed, we developed a reward-biased simple RT task in rats that used differential reward expectations to modulate motivational salience (Figure 1A, Figure S1). Specifically, each trial started with a light signal that instructed rats to enter a nosepoke fixation port where they maintained fixation until an auditory stimulus instructed them to collect a water reward in the adjacent reward port. In the fixation port, rats heard, with equal probability and randomly across trials, either a sound predicting a large reward (S-Large), a different sound predicting a small reward (S-Small), or no sound and no reward (Catch). S-Large and S-Small were chosen to be clearly discriminable (white noise versus clicker) and presented at a suprathreshold level (80 dB for 2 s) to minimize sensory detection and discrimination effort. After sound onset, rats exited the fixation port quickly and moved to the adjacent reward port in almost all S-Large (99.8%) and S-Small (99.4%) trials, and only did so occasionally in Catch trials (3.8%). This response pattern confirmed that rats treated both S-Large and S-Small as motivationally salient stimuli. The latency between sound onset to fixation port exit, defined as RT, reflected the speed of the initial decision-making process in response to motivationally salient sounds and is the focus of our study.

While both S-Large and S-Small predicted reward in the same port and therefore commanded the same behavioral response without the need of a choice, well-trained rats automatically responded faster in S-Large than in S-Small trials (Figure 1B, Figure S1), indicating that the stimulus paired with larger reward was motivationally more salient. The modulation of RT between S-Large and S-Small trials continued to grow and did not reach asymptotic level after 10 training sessions (Figure 1C, Figure S1). Following the reversal of sound-reward contingency, it took rats on average three sessions to reverse their RT bias and began to show faster RT toward the new S-Large (Figure 1C, Figure S1). Thus, the reward-biased simple RT task provided a large dynamic range of RT modulation between S-Large and S-Small trials.

The reward-biased simple RT task was designed to minimize several variables that also affect RT: First, the influence of trial-by-trial variation in motivational state such as fatigue and satiety was minimized by requiring rats to initiate each trial with the same nosepoke fixation response. Second, the influence of choice—that is, choosing between different response options—on RT was minimized because both S-Large and S-Small signaled reward in the same port. Third, the influence of stimulus uncertainty and sensory decision-making process on RT was minimized by using sounds well above the detection threshold. Finally, this task design ensured that any other variable that affects RT at the time of sound onset, such as temporal expectation of stimulus onset or the spontaneous activity state of the neural network, should similarly affect both S-Large and S-Small trials. These behavioral and neuronal variabilities not directly controlled by the experimenters are collectively labeled as intrinsic noise of the decision-making process in the current study and should be equivalent between S-Large and S-Small trials. As a result, the RT difference between S-Large and S-Small trials must arise from the difference in the properties of the stimulus, such as the associated motivational salience. Therefore, the reward-biased simple RT task serves to provide the necessary simple behavioral context to reveal the quantitative relationship between BF motivational salience signal and RT, while minimizing the influence of other variables.

**Figure 2:**
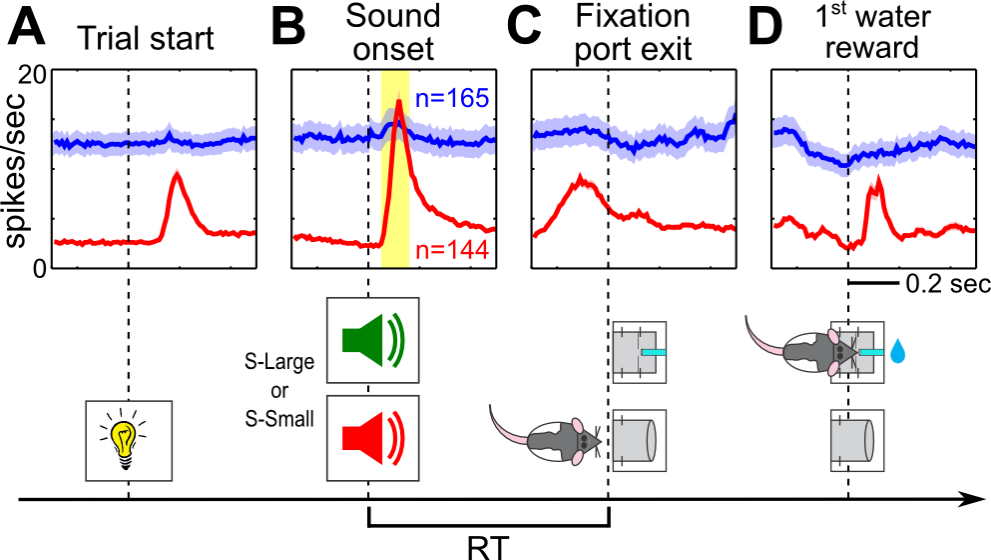
Phasic bursting response of BF neurons encodes motivational salience and precedes RT. BF population PSTH to trial start light signal (A), sound onset (B), fixation port exit (C), and the first drop of water reward (D). The population PSTH (mean ± sem) for BF bursting neurons (red, *n* = 144) and all other BF neurons (blue, *n* = 165) recorded from six rats in 40 sessions. The yellow shaded area indicates the {50, 160} ms window used to calculate BF bursting amplitude. BF bursting neurons also showed bursting responses to trial start signal and reward. The bursting response to sound onset largely dissipated before RT.

We first investigated whether BF motivational salience signal occurred early enough to modulate RT and decision speed. In six rats recorded over 40 sessions, we recorded 309 well-isolated single units in the region of the BF where cortically projecting BF neurons are located (Figure S2) [28]–[30]. Of these BF neurons, 47% (144/309) showed prominent bursting responses to sound onset and were classified as BF bursting neurons (Figure 2B, Figure S3). The same neurons also showed bursting responses to the trial start light signal (Figure 2A) and reward delivery (Figure 2D), consistent with the encoding of motivational salience as we previously reported [16]. This bursting response began at 50 ms after sound onset and peaked at 120 ms (Figure 2B), considerably earlier than almost all RTs in all behavior sessions (Figure 1B), and largely dissipated when rats exited the fixation port (Figure 2C). Therefore, BF motivational salience signal occurred early enough in the decision process to modulate the fixation port exit RT.

**Figure 3:**
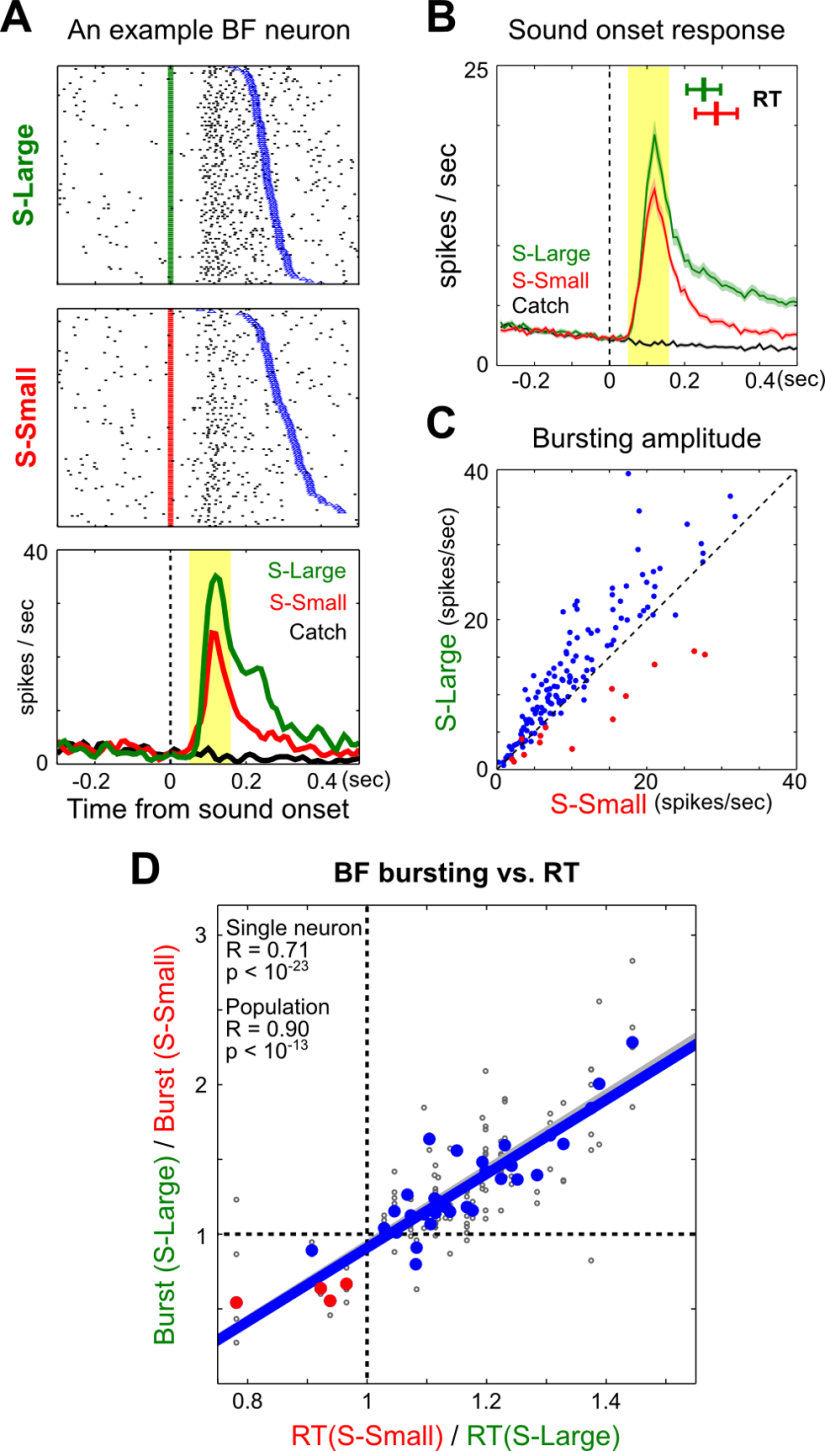
BF bursting amplitude predicts RT modulation between S-Large and S-Small trials. (A) Bursting responses of one representative BF neuron to S-Large and S-Small onset. Individual trials in raster plots were aligned to sound onset and sorted by RT (blue). (B) Population PSTH (mean ± sem) for BF bursting neurons (*n* = 144) showed stronger bursting to S-Large than to S-Small. The mean RTs for the corresponding trials were indicated in the inset (mean ± std, *n* = 40 sessions). (C) Scatter plot of the mean bursting amplitude for each BF bursting neuron in S-Large versus S-Small trials from one session. Each dot represents one BF bursting neuron (*n* = 144), with red dots representing neurons recorded during the first three sessions after reversal (*n* = 14). (D) Correlation between BF bursting amplitude modulation and mean RT modulation in one session, each calculated as a ratio between S-Large and S-Small trials. Results plotted separately for individual BF bursting neurons (gray), as well as for the entire bursting population per session during the first three reversal sessions (red) or afterwards (blue). Between S-Large and S-Small trials in a session, BF bursting strength was strongly correlated with the modulation of mean RT.

Next, we investigated whether the strength of motivational salience, defined as the amplitude of the BF bursting response, was correlated with decision speed. A typical example of salience-encoding BF neuron is shown in Figure 3A, which illustrates how these neurons respond to both S-Large and S-Small onset at a fixed latency relative to stimulus onset and robustly in each trial in well-trained animals. This example neuron also illustrates the common finding that the strength of BF bursting response was stronger toward S-Large than toward S-Small onset (Figure 3B,C). This is consistent with the intuition that pairing with the larger reward should endow a stronger motivational salience to S-Large than to S-Small.

The critical comparison was whether the RT modulation between S-Large and S-Small trials was correlated with the modulation of BF bursting amplitude between these two trial types. We found that the modulation of BF bursting amplitude at both single neuron and population level were highly correlated with the modulation of mean RT between S-Large and S-Small trials in a session (Figure 3D, Figure S4). The modulation of BF bursting tracked the modulation of mean RT even during the first three sessions of reversal learning when the RT bias had not been updated to reflect expected reward (red dots in Figure 3D). These findings provide key support of our hypothesis that the difference in decision speed between the two trials types was mostly driven by the difference in their associated motivational salience, encoded in the BF. Since S-Large, S-Small, and Catch trials were randomly intermingled in a session, BF motivational salience signal is coupled with RT on a single trial basis.

**Figure 4:**
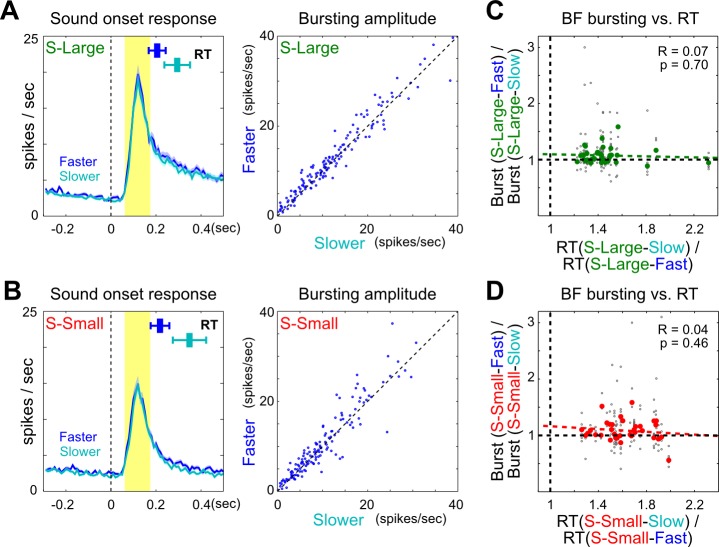
No modulation of BF bursting amplitude within a trial type. (A and B) Population PSTHs (mean ± sem) and scatter plots of bursting amplitude for all BF bursting neurons in faster (blue) and slower (cyan) RTs within S-Large (A) and S-Small (B) trials. Convention as in Figure 3B–C. There was little modulation of BF bursting amplitude within a trial type. (C–D) Correlation between BF bursting amplitude modulation and mean RT modulation between faster and slower RTs within S-Large (C) and S-Small (D) trials. Convention as in Figure 3D.

Having demonstrated the strong coupling between BF bursting amplitude and RT modulation, we further investigated whether this coupling was similarly present within, and not just between, S-Large and S-Small trials. We reasoned that if BF bursting amplitudes similarly predicted RT within the same trial type, the largest difference in BF bursting amplitude should be observed between the fastest and slowest trials. However, we found that there was little modulation of BF bursting strength even between these trials that had the largest RT difference within each trial type (Figure 4A,B, Figures S5 and S6), and the modulation of BF bursting amplitude did not correlate with RT modulation (Figure 4C,D). These results suggest that the trial-by-trial RT variability within each trial type was not correlated with the moment-to-moment fluctuation of BF motivational salience signal across trials. Instead, the within-trial-type RT variability likely reflected the contribution from the intrinsic noise of decision-making process in the presence of highly similar BF bursting amplitude and behavioral states across trials.

**Figure 5:**
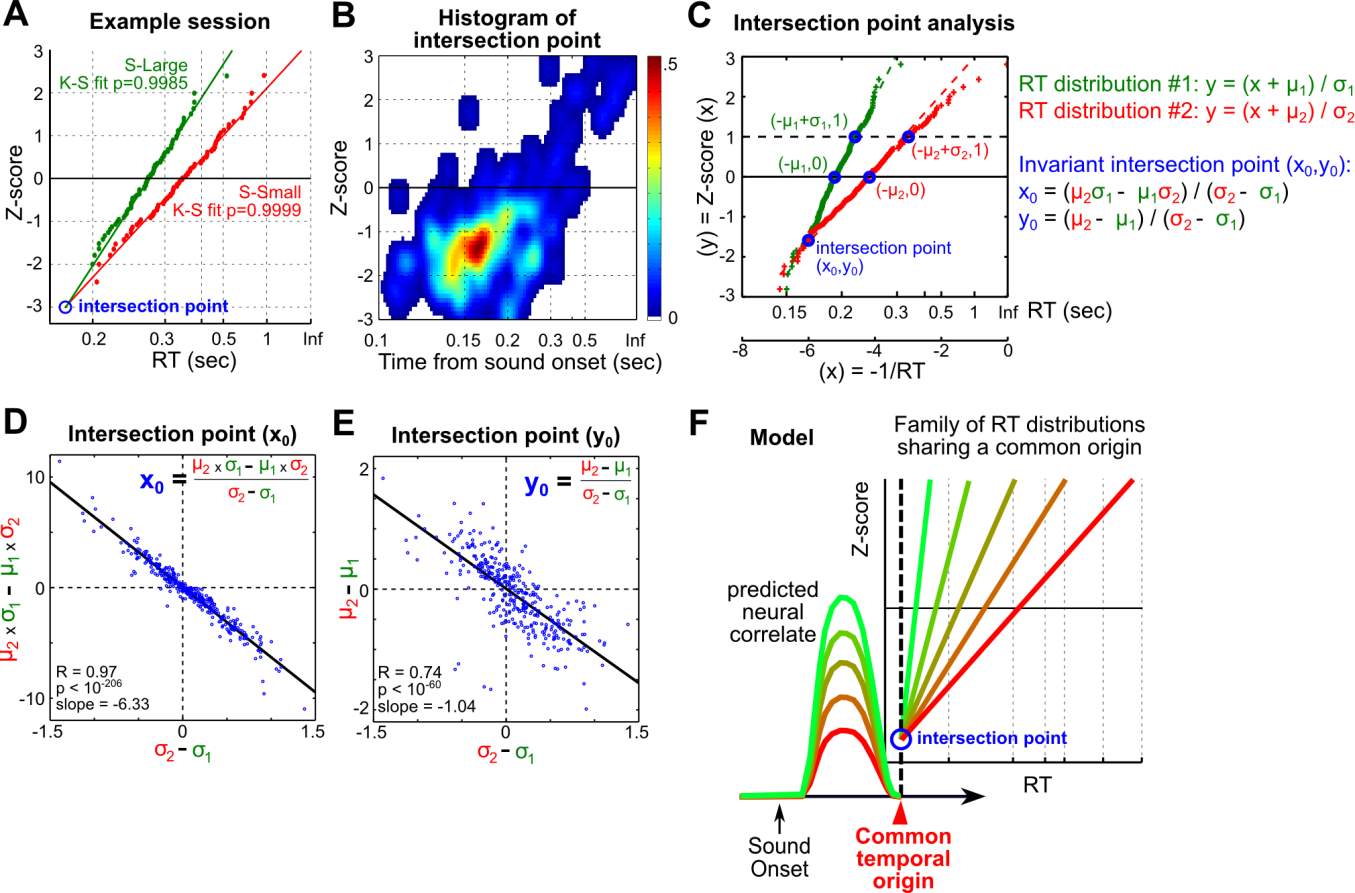
RTs are organized as a family or recinormal distributions swiveling against a fix time point. (A) A representative session shows that RTs in S-Large and S-Small trials were recinormally distributed and can be transformed into a straight line by plotting 1/RT versus its *z*-score. Each dot represents RT from one trial. The intersection point of the two RT distributions is indicated by the blue circle. (B) 2-D histogram of the intersection point across sessions shows that the two RT distributions typically intersected around 160 ms. (C) Schematic of the intersection point analysis. Transforming the two RT distributions into straight lines by plotting −1/RT versus its *z*-score predicts novel invariant relationships between the intersection point (x_0_, y_0_) and parameters (μ_1_, σ_1_) and (μ_2_, σ_2_). (D) The *x*-coordinate of the intersection point is expressed as (μ_2_σ_1_−μ_1_σ_2_)/(σ_2_−σ_1_). The numerator and denominator are plotted on the *y*- and *x*-axis, respectively, along with the linear fit. Each dot is derived from RT distributions in one session (*n* = 339, 16 rats). (E) The *y*-coordinate of the intersection point is expressed as (μ_2_−μ_1_)/( σ_2_−σ_1_). Convention as in (D). (F) These data support the model that RT distributions swivel against a fixed time point, and predict that this family of RT distributions can be generated by a single neural mechanism whose activation level sets the parameters of RT distributions.

To better understand the nature of the within-trial-type RT variability, we noted its similarity with the considerable RT variability in humans when sensory ambiguity is reduced to a minimum, which has long been proposed to reflect the contribution of intrinsic noise in the decision-making process [31]–[33]. In humans, RT variability to suprathreshold sensory stimuli like the ones used in the current study, but not RT variability toward ambiguous sensory inputs, is highly structured and best described by the recinormal distribution [31],[32],[34]. Recinormal RT distribution means that the reciprocal of RT (1/RT) is normally distributed and that the RT distribution can be transformed into a straight line by plotting 1/RT versus its *z*-score (Figure S7). Therefore, we tested whether recinormality can be extended to the rat and found that the within-trial-type RT variability was well-described by the recinormal distribution (Figure 5A, Figure S7). This finding supports the universality of recinormal RT distribution across species and response modalities, and supports the hypothesis that within-trial-type RT variability likely reflected the contribution of intrinsic noise in the decision-making process.

The empirical observation that the within-trial-type RT variability is well described by the recinormal distribution suggests that the recinormal distribution provides an ideal quantitative framework to understand the two sources of RT variability in our study. Specifically, we hypothesize that the variability (σ) of the underlying normal distribution provides an estimate of the influence from intrinsic noise on RT, whereas the mean speed (μ) of the normal distribution captures the between-trial-type RT variability modulated by the BF motivational salience signal. Therefore, the quantitative relationship between μ and σ parameters of S-Large and S-Small RT distributions should provide insights on the relationship between the two sources of RT variability, and by extension the relationship between BF motivational salience signal and intrinsic noise of the decision-making process.

Interestingly, we observed that S-Large and S-Small RT distributions in a session often intersected near their respective fastest RT at a fixed time point around 160 ms (Figure 5B), suggesting that all RT distributions swivel against a fixed time point. To further investigate the consequences of swiveling against a fixed time point, we solved the linear equations for the two RT distributions with parameters (μ1, σ1) and (μ2, σ2) under the constraint of a fixed intersection point (Figure 5C). The fixed swivel point predicted two invariant relationships between parameters (μ1, σ1) and (μ2, σ2), which revealed previously unknown and exceedingly strong correlations between μ and σ parameters of RT distributions (Figure 5D,E). These novel correlations indicate that RT distributions with a larger mean speed μ (faster RTs) are associated with a shrinking variability σ. When μ approaches the theoretical limit (vertical black dotted line in Figure 5F), the mean RT approaches its fastest limit equivalent to the swivel point ∼160 ms while the RT variability (σ) shrinks to zero. This extreme scenario underscores the general finding that the RT variability (σ) does not scale proportionally with RT (1/μ), as would be expected from Weber's law, but in fact shrinks much faster. In the broader context of between- versus within-trial-type RT variability, the novel correlations between μ and σ revealed here suggest that these two sources of RT variability are tightly co-regulated and not independent. The magnitude of within-trial-type RT variability (σ), reflecting the magnitude of contribution from intrinsic noise, is actively suppressed in faster RT distributions with higher speed (μ) and stronger BF motivational salience signals.

The co-regulation of μ and σ parameters of RT distributions further suggests that each RT distribution can be determined with only one free parameter instead of two. From this perspective, the organization of RTs in our study can be viewed as a family of RT distributions swiveling against a fixed time point around 160 ms, with only one degree of freedom (Figure 5F). As such, our analysis predicts that this family of RT distribution can be generated by one single neural mechanism with three predicted properties essentially those of the BF motivational salience signal. First, this neural mechanism should occur before the fixed swivel point (∼160 ms) like the BF bursting response (Figure 2). Second, the activity of this neural mechanism should determine the speed (μ) and variability (σ) parameters of RT distributions, similar to how BF bursting amplitude is correlated with the mean RT (Figure 3). Third, it predicts that the same speed (μ) and variability (σ) parameters should be shared by all trials within a recinormal RT distribution regardless of whether RT is fast or slow, similar to the invariant BF bursting amplitude across all trials within a trial type (Figure 4). Therefore, these results support that BF motivational salience signal serves as a neural correlate of RT distribution parameters.

**Figure 6:**
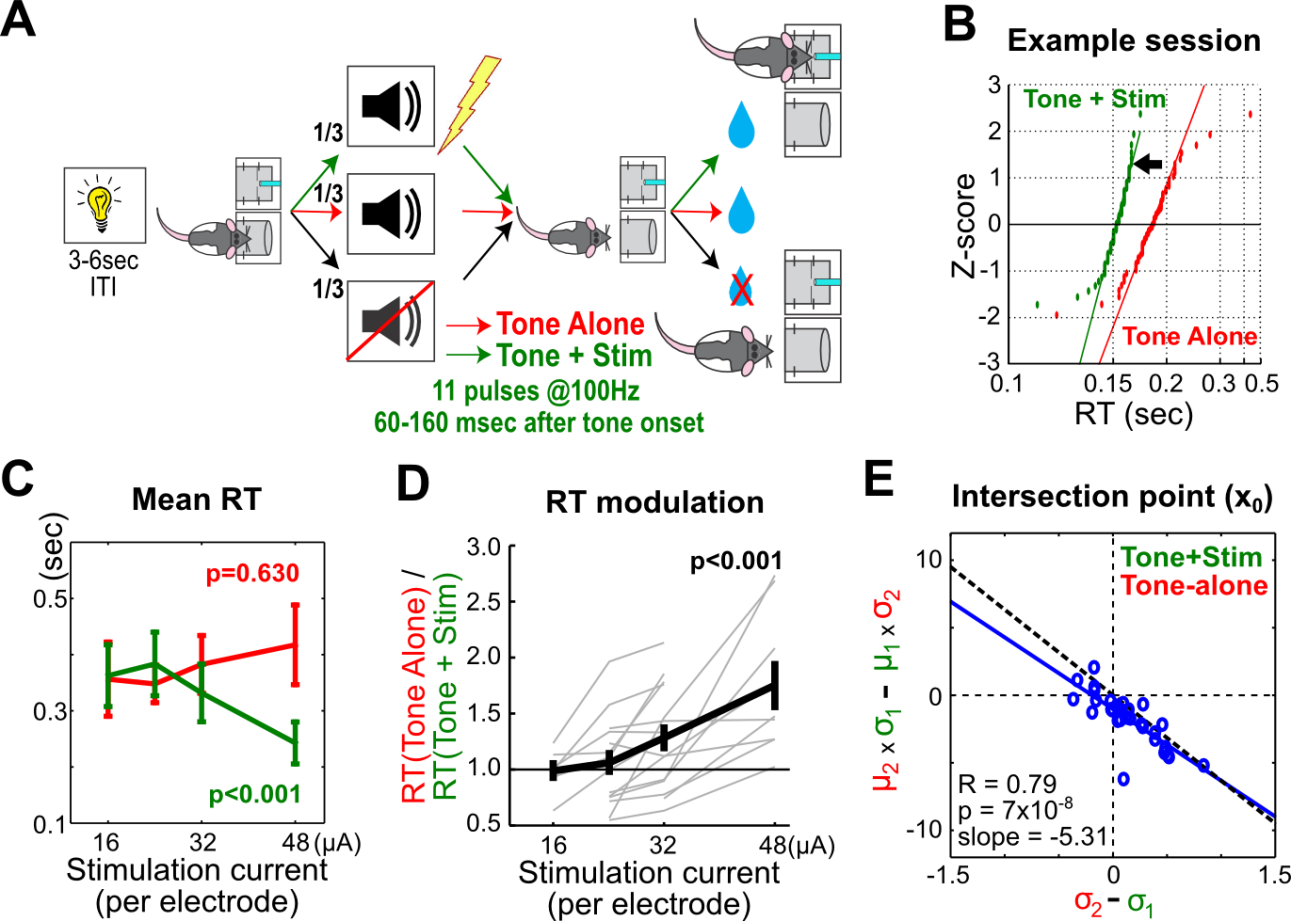
Augmenting BF bursting strength via BF electrical stimulation leads to faster and more precise RTs. (A) Schematic of the BF stimulation task. An identical 6 kHz tone was presented either paired with or without BF electrical stimulation delivered during the BF bursting window. Both trial types led to the same reward amount. (B) An example session shows that BF electrical stimulation led to a faster RT distribution compared to nonstimulated tone-alone trials. Convention as in Figure 5A. (C) Increasing BF stimulation current led to faster RTs (mean ± sem) in stimulated trials but no change of RT in nonstimulated trials (linear mixed model, *n* = 7 rats, 44 sessions). (D) Stronger stimulation current led to stronger RT modulation between stimulated and nonstimulated trials (linear mixed model). Data from individual animals plotted as gray lines, with the population mean ± sem in black. (E) BF stimulation also produced more precise RTs while largely preserving the coupling between μ and σ parameters of RT distributions as seen in Figure 5D. The blue line represents the linear regression, whereas the black dotted line represents the linear regression from Figure 5D for comparison. See Figure S9 for more details.

Finally, to investigate whether the observed coupling between the strength of BF motivational salience signal and the speed and variability of a RT distribution represents a causal relationship, we tested the prediction that augmenting the strength of BF motivational salience signal via electrical stimulation should produce faster and more precise RT distributions. The experimental setting was the same as the reward-biased simple RT task, except that S-Large and S-Small sounds were replaced by a common 6 kHz tone in both trial types and either paired with or without BF microstimulation (Figure 6A, Figure S8). The BF electrical stimulation was precisely timed to coincide with the tone-induced bursting response as a way to artificially augment the BF bursting amplitude. Under this protocol, RTs in the stimulated trials indeed became faster relative to nonstimulated control trials (Figure 6B–D). This RT difference grew larger as the stimulation current increased, consistent with the observation that greater bursting amplitudes were associated with faster RT distributions. Furthermore, BF electrical stimulation also produced more precise RTs such that the coupling between μ and σ parameters of RT distributions remained largely unchanged (Figure 6E and Figure S9). This result therefore supports a causal role of the BF motivational salience signal in determining both the speed and variability of RT distributions.

## Discussion

This study examined how motivational salience modulates decision speed. Our results provide strong support that the motivational salience signal in the BF, encoded by the phasic bursting response [16], is a major determinant of decision speed. We found that the BF bursting response took place early in the decision-making process and occurred early enough to modulate RT in the reward-biased simple RT task (Figure 2). RT variability in this task can be partitioned into two distinct sources, with the between-trial-type RT modulation tightly correlated with the strength of BF motivational salience signal (Figure 3), whereas the within-trial-type RT variability was unrelated to the BF motivational salience signal and likely reflected the intrinsic noise of the decision-making process (Figure 4). Analysis of the organization of RTs using recinormal distribution revealed that these two sources of RT variability were highly coupled, where RT distributions with fast mean RTs were associated with shrinking RT variability (Figure 5). Artificially augmenting the BF bursting amplitude via electrical stimulation increased decision speed as a function of stimulation current amplitude and also reduced variability, consistent with a causal relationship (Figure 6). Together, these results support the hypothesis that the BF motivational salience signal increases decision speed and also suppresses the contribution of intrinsic noise on RT variability.

Although the correlation between BF motivational salience signal and RT was fully predicted based on the literature [13]–[16], this is the first study, to our knowledge, to demonstrate the quantitative relationship between RT and a neural correlate of motivational salience defined independently of RT. BF bursting amplitude and RT were correlated on a single trial basis because the three trial types (S-Large, S-Small, and Catch) were intermingled and randomly presented in the session. Therefore, BF bursting amplitude fluctuated significantly across trials (of different trial types) and provided a good predictor of the RT on that trial. Within the same trial type, however, BF bursting amplitude remained highly consistent across trials, reflecting the highly similar behavioral and motivational states.

The reward-biased simple RT task was designed to minimize the influence of other variables on RT such that RT variability was mainly driven by motivational salience and by the intrinsic noise of the decision-making process. To determine the respective contributions of BF motivational salience signal and intrinsic noise on RT, our approach was to systemically vary the contribution from these two sources of RT variability. When the BF bursting amplitude was held constant within a trial type (Figure 4), the trial-by-trial RT variability was unrelated to the moment-by-moment fluctuation of BF motivational salience signal and therefore reflected the contribution from intrinsic noise of decision-making. When BF bursting amplitude was modulated between S-Large and S-Small trials (Figure 3), BF motivational salience signal accounted for most of the between-trial-type RT variability. The contribution of BF bursting on RT was further supported by the responses to the trial start light signal (Figure S10), in which the large fluctuation of BF bursting amplitude across trials was the main contributor to RT variability and was associated with clearly visible trial-by-trial coupling between BF bursting amplitude and overall response latency. Therefore, the lack of correlation between BF bursting amplitude and RT within a trial type does not mean that BF bursting is not correlated with RT. Rather, it reflects a principled approach to estimate the contribution of intrinsic noise on RT variability.

Our findings support that the recinormal distribution provides a quantitative framework, across species and response modalities, to describe the contribution of intrinsic noise on RT variability. Although recinormal RT distributions have been described to swivel against fix points under other behavioral contexts [35]–[38], our finding is the first, to our knowledge, to describe that RT distributions can swivel against a fixed time point near their respective fastest RT (Figure 5). This novel swiveling pattern revealed previously unknown correlations between the speed (μ) and variability (σ) parameters of recinormal RT distributions, and suggests that the contribution of intrinsic noise can be actively suppressed to near zero RT variability in the presence of a fast mean RT and strong BF motivational salience signal. This analysis also suggests that RT in a single trial is jointly determined by the parameters (μ and σ) of the recinormal distribution, and by the stochastic intrinsic noise that randomly draws from the recinormal RT distribution. Determining the parameters of the recinormal distribution therefore represents a previously unrecognized yet essential step in the decision-making process, which determines both the decision speed and its precision and is dictated largely by the BF motivational salience signal.

Our study is also the first, to our knowledge, to demonstrate that artificially increasing BF bursting amplitude through BF electrical stimulation was sufficient to produce faster and more precise RTs (Figure 6). Because stimulated and nonstimulated trials (as well as catch trials) were randomly intermingled in the session, our results suggest that the effect of BF electrical stimulation was transient and did not affect RT in subsequent trials. This is consistent with the trial-by-trial coupling between BF bursting amplitude and RT (Figure 3). We believe that this transient influence on RT by BF electrical stimulation is more consistent with a transient increase of the motivational salience associated with the stimulus, and less consistent with an increase in general arousal, which should fluctuate at a much slower time scale but not in single trials.

The current study replicated and extended our previous findings linking BF bursting response to motivational salience. We found that BF bursting neurons not only responded to multiple motivationally salient stimuli in the reward-biased simple RT task (Figure 2), their bursting response also reflected the influence of other behavioral variables on motivational salience. For example, when rats were not required to maintain fixation, BF bursting amplitude in response to the trial-start light signal showed large fluctuation across trials (Figure S10), presumably reflecting the influence of fluctuations in arousal, fatigue, or satiety on motivational salience. This fluctuation of BF bursting amplitude was coupled with response latency on a trial-by-trial basis (Figure S10). Furthermore, in the early sessions of reversal learning when the RT bias had not been updated to reflect expected reward, BF bursting remained tightly coupled with RT modulation (Figure 3D). These data provide further support that BF bursting amplitude reflects motivational salience and is tightly coupled with decision speed. The role of BF motivational salience signal in the learning process is an important question that needs further investigation in future studies.

The widespread spatial distribution of BF bursting neurons is consistent with the location of cortically projecting BF neurons as revealed by placing retrograde tracers in the prefrontal cortex (Figure S2) [28]. The cortically projecting BF neurons are not restricted to a specific subregion in this area, but spread across multiple subregions, including the ventral part of globus pallidus (GP), ventral pallidum (VP), substantia innominata (SI), nucleus basalis of Meynert (NBM, or B), magnocellular preoptic nucleus (MCPO), and horizontal limb of the diagonal band (HDB), but not in the adjacent hypothalamic region lateral preoptic area (LPO) [28]. Furthermore, a recent study [39] shows that individual BF neurons tend to project to multiple subregions in the frontal cortex, unlike single neurons in other neuromodulatory systems, which tend to project to one single subregion in the frontal cortex. This finding suggests that the exact location of BF neurons, as well as the location of BF stimulation electrode, is less critical because the activity of any subset of cortically projecting BF neurons likely provide similar modulation of the entire frontal cortex. Previous studies have shown that the salience-encoding BF neurons represent a physiologically homogeneous group of noncholinergic BF neurons, which, unlike cholinergic BF neurons, do not change their mean firing rates between awake and sleep states [16],[21]. Given that the neurochemical identity of BF bursting neurons remains to be determined, electrical stimulation is the best available technique to ensure the activation of BF bursting neurons for testing causality. One appealing possibility is that salience-encoding BF neurons may correspond to the cortically projecting GABAergic BF neurons, which represent an anatomically prominent projection to the cerebral cortex [28]–[30] and primarily innervate on interneurons in the cortex [40],[41]. The activation of these long-range projecting GABAergic BF neurons should transiently inhibit cortical interneurons, releasing cortical pyramidal neurons from inhibition, leading to an increase in response gain modulation and ultimately resulting in faster decision speed.

The coupling with faster and more precise decision speed demonstrated in this study adds to the functional significance of this anatomically prominent [28]–[30] yet previously neglected neuronal population in the BF [42],[43]. Dissecting the neural circuit-level mechanisms of the BF motivational salience signal will have important translational implications. Dysregulation of motivational salience coupled with decreased decision speed are well documented in schizophrenia [8],[9],[44] and depression [4],[5],[45]. Significant decreases in decision speed also represent a key feature in dementia [6],[7] and cognitive aging [11],[12]. Although the dysregulation of motivational salience has traditionally implicated dopamine neurons and the literature on dementia and cognitive aging has largely focused on cholinergic BF neurons, our study points to a novel and previously neglected candidate mechanism in these conditions. We propose that the decline of decision speed in some of these conditions may result from either the functional impairment of the BF motivational salience system or a disrupted cortical-BF interaction.

## Materials and Methods

### Ethics Statement

All experimental procedures were conducted in accordance with the National Institutes of Health (NIH) Guide for Care and Use of Laboratory Animals and approved by the National Institute on Aging Animal Care and Use Committee.

### Subjects

Twenty-two male Long Evans rats (Charles River, NC), aged 3–6 mo and weighing 300–400 grams at the start of the experiment, were used for this experiment. Sixteen of the 22 rats were trained in the reward-biased simple RT task, with 6/16 of this group undergoing surgery for chronic neuronal activity recording. Seven rats were used in the electrical microstimulaiton experiment, including one of the six rats used for neuronal activity recording, and the other 6/22 rats were used exclusively for the electrical stimulation experiment.

Rats were housed under 12/12 day/night cycle with *ad libitum* access to rodent chow and water in environmentally controlled conditions. During training and recording procedures, rats were mildly water restricted to their 90% weight and were trained in a daily session of 60–90 min in length, 5 d a week. Rats received 15 min water access at the end of each training day with free access on weekends.

### Apparatus

Twelve plexiglass operant chambers (11″L×8 ¼ ″W×13″H), custom-built by Med Associates Inc. (St. Albans, VT), were contained in sound-attenuating cubicles (ENV-018MD) each with an exhaust fan that helped mask external noise. Each chamber was equipped with one photo-beam lickometer reward port (CT-ENV-251L-P) located in the center of the front panel, with its sipper tube 7.5 cm above the grid floor. Two infrared (IR) sensors were positioned to detect reward port entry and sipper tube licking, respectively. Water reward was delivered through a custom-built multibarrel sipper tube. The delivery system was controlled by pressurized air and each solenoid opening (10 ms) was calibrated to deliver a 10 µl drop of water. The reward port was flanked by two nosepoke ports (ENV-114M), located 6.6 cm to each side and 5.9 cm above the grid floor. Each nosepoke port was equipped with one IR sensor to detect port entry. Only the right nosepoke port was used in behavioral training as the fixation port, while the left nosepoke port was inactive.

Each chamber was equipped with two ceiling-mounted speakers (ENV-224BM) to deliver auditory stimuli, and two stimulus lights (ENV-221) positioned above the reward port in the front panel. Behavior training protocols were controlled by Med-PC software (Version IV), which stored all event timestamps at 2 ms resolution and also sent out TTL signals to neurophysiology recording systems to register event timestamps.

### Behavioral Training

#### Shaping protocol

All rats (*n* = 22) were initially trained to respond to 6 kHz tone (2 s, 80 dB) in the operant chamber to receive three drops of water in the reward port, delivered starting at the third lick. Only trials with the third lick completed within a 3 s reward window were defined as successful go response and rewarded. Subsequently, rats were shaped to nosepoke in the fixation port and maintain fixation until tone presentation. Four different foreperiods (0.35, 0.5, 0.65, and 0.8 s) were used, pseudorandomly across trials, to minimize temporal expectation of stimulus onset. Tone was delivered in 2/3 of trials (rewarded), whereas no sound was delivered in the other 1/3 trials (catch trials with no reward). Early fixation port exit before the foreperiod resulted in no reward delivery. The inclusion of catch trials ensured that tone onset was the most reliable predictor of reward and that rats did not employ a timing strategy for responding.

The intertrial interval (ITI) was 3–6 s, signaled by a white stimulus light. The offset of the light thus served as the trial start signal, indicating that fixation port entry could now lead to tone presentation. Premature fixation port entry and premature licking both resulted in resetting the ITI timer. After rats reached asymptotic performance after 1–2 mo of training, 16 rats were subsequently trained in the reward-biased simple RT task, whereas the other six were used exclusively in the electrical microstimulation experiment. Note that the shaping protocol was essentially the same as the one used for electrical microstimulation (Figure 6A), except that no BF electrical stimulation was delivered.

#### Reward-biased simple RT task

The reward-biased simple RT task was the same as the shaping protocol except that rats were presented with three different conditions with equal probability in the fixation port: (1) white noise, (2) 100 Hz clicker sound, or (3) catch trials (Figure 1A). The two auditory stimuli were chosen to be clearly discriminable and presented at a suprathreshold level (2 s, 80 dB) to minimize sensory detection and discrimination effort. The two sounds were associated with either 1 versus 4 or 4 versus 1 drops of water in a session. Each rat was trained on a particular reward amount schedule over several sessions until a clear RT difference between S-Large and S-Small trials emerged. The animals then underwent serial reversal learning where the reward amount schedule was reversed, such that the sound previously paired with a small reward was paired with a large reward, and vice versa (Figure S1E–H). Behavior and neural data were collected during both the initial learning as well as during subsequent serial reversal learning of the sound-reward association. Because the sound associated with large reward was counterbalanced across animals and across serial reversal learning phases, the sound predicting the larger reward will be referred to as S-Large, whereas the sound predicting the smaller reward was referred to as S-Small.

For the final behavior analysis, only sessions meeting the following criteria were included: (1) the animal completed at least 50 trials per trial type; (2) mean RT in both S-Large and S-Small trials must be faster than 500 ms; and (3) the proportion of catch trials with fast RT (less than 300 ms) must be less than 10%, ensuring a low response propensity in the absence of sound. A total of 431 sessions met these criteria. Of these sessions, 79% (339/431) were well described by the recinormal distribution (Kolmogorov–Smirnov, or K-S, test *p* value≥0.6 for both S-Large and S-Small trials in Figure S7) and were used for the final analysis. In these sessions, rats completed on average 111±46 (mean ± std) trials per trial type (on average 333 total trials in a session), with 227±65 ms RT (mean ± std) to S-Large, 260±77 ms RT (mean ± std) to S-Small, and had 4.3%±2.7% (mean ± std) catch trials with fast RT (less than 300 ms).

The strategic positioning of three IR sensors partitioned the entire response time (between sound onset to reward collection) into four epochs: (1) sound onset to fixation port exit, (2) fixation port exit to reward port entry, (3) reward port entry to the first lick, and (4) first lick to the third lick (the first drop of water reward). The RT measure, which corresponds to the first response time epoch, was the segment of the entire response trajectory showing the largest behavioral difference between S-Large and S-Small trials (Figure S1). This indicates that rats were able to make rapid behavioral discrimination between S-Large and S-Small trials.

Behavioral and neural data were analyzed using custom scripts in Matlab 2012a (Mathworks, MA), and statistical tests were analyzed using SPSS (Version 20, IBM Corp).

### RT Analysis

#### Recinormal RT distribution

Previous studies have established that RTs in response to noisy or ambiguous sensory stimuli—that is, conditions that require considerable sensory evidence accumulation—are well described by a random-walk diffusion model [34]. On the other hand, simple RTs in response to suprathreshold stimuli are best characterized by the recinormal distribution [34]. This dichotomy likely represents two distinct stages in the decision-making process [34].

The recinormal property of RT distributions means that the reciprocal of RT (1/RT) is normally distributed, which can be fully characterized by its mean (μ) and standard deviation (σ). Because a normal distribution can be transformed into a straight line when plotted against its *z*-score transformation, a recinormal RT distribution can be transformed into a straight line by plotting 1/RT versus its *z*-score (Figure S7). As a convention to keep faster RTs to the left of the plot, −1/RT was used instead of 1/RT as the *x*-coordinate. K-S test was used to compare the empirical RT distribution with the fitted recinormal RT distribution, and to determine the best estimate of the μ and σ parameters that produced the minimal *p* values for the K-S test (Figure 5A, Figure S7).

The empirical observation of recinormal RT distributions has led to the development of the Linear Approach to Threshold with Ergodic Rate (LATER) model [31], which posits that recinormal RT distributions can be generated by a simple stochastic neural process that accumulates activity at a constant rate until reaching a decision threshold. By arbitrarily setting the baseline neural activity as 0 and the decision threshold as 1, the constant rate of neural activity accumulation (or rise rate) corresponds to the normally distributed 1/RT∼N(μ,σ^2^).

### Stereotaxic Surgery and Electrode

After reaching asymptotic behavioral performance, rats were taken off water restriction for at least 3 d before undergoing stereotaxic surgery for chronic electrode implants. Rats were anesthetized with isoflurane (2%–4% isoflurane induction followed with 1%–2% maintenance) and received atropine (0.02–0.05 mg/kg, i.m.) to reduce respiratory secretion. The incision area was shaved and cleaned with betadine, and injected first with local anesthetic (1% mepivacaine HCl solution). Ophthalmic ointment was applied to prevent corneal dehydration. A heating pad was used to maintain body temperature at 37°C. Rats were placed in a stereotaxic frame (David Kopf Instrument, CA) fitted with atraumatic earbars.

Multiple skull screws were inserted first, with one screw over the cerebellum serving as the common electrical reference and a separate screw over the opposite cerebellum hemisphere serving as the ground. A custom-built 32-wire multi-electrode moveable bundle was implanted into bilateral BF. The electrode consisted of two moveable bundles, each containing 16 polyimide-insulated tungsten wires (California Fine Wire, CA) ensheathed in a 28-gauge stainless steel cannula and controlled by a precision microdrive. Eight of the wires in a bundle were 38 µm in diameter and the other eight were 16 µm diameter, with 0.1–0.3 MΩ impedance measured at 1 kHz (niPOD, NeuroNexusTech, MI). The two cannulae of the electrode were precisely positioned to target the BF on both hemispheres at AP –0.6 mm, ML ±2.25 mm relative to Bregma [46]. During surgery, the cannulae were lowered to DV 6–6.3 mm below cortical surface using a micropositioner (Model 2662, David Kopf Instrument), and the electrodes were advanced to 7 mm below the cortical surface. After reaching target depth, the electrode and screws were covered with dental cement (Hygenic Denture Resin). Rats received acetaminophen (300 mg/kg, oral) and topical antibiotics after surgery for pain relief and prevention of infection. Water restriction and behavioral training resumed 7–10 d after surgery. Cannulae and electrode tip locations were verified with cresyl violet staining of histological sections at the end of the experiment and compared with reference anatomical planes [46]. All electrodes were found at expected positions (Figures S2 and S8).

### Recording

Each recording session lasted 60–90 min. At the conclusion of each recording session, BF electrodes were advanced at least 100 µm and 3–7 d elapsed before the next recording session. One recording session at each electrode depth was included in the final analysis and therefore sampled distinct BF single neuron ensembles. A total of 309 BF single units were recorded from 40 sessions in six rats, at DV 7.1–8.3 mm below the cortical surface.

Electrical signals were referenced to a common skull screw placed over the cerebellum. Electrical signals were filtered (0.03 Hz–7.5 kHz) and amplified using Brighton Omnetics or Cereplex M digital headstages and recorded using a Neural Signal Processor (Blackrock Microsystems, UT). Single unit activity was further filtered (250 Hz–5 kHz) and recorded at 30 kHz. Spike waveforms were sorted offline using OfflineSorter (Plexon Inc, TX). Only single units with clear separation from the noise cluster and with minimal (<0.1%) spike collisions (spikes with less than 1.5 ms interspike interval) were used for further analyses. Additional cross-correlation analysis was used to remove duplicate units recorded simultaneously over multiple electrodes.

### Identification of BF Bursting Neurons

Peri-stimulus time histograms (PSTHs) were generated for each BF single neuron against each event using a 10 ms bin. To determine whether each BF neuron significantly responded to sound onset, we subtracted the neuronal response in catch trials from the response in sound-present trials for each neuron. This was necessary because many BF neurons changed their activity during the foreperiod while waiting for sound onset inside the fixation port. This subtraction procedure removed the nonstationary baseline before sound onset (see Figure S5) and allowed us to ask whether BF neurons truly responded to sounds. To determine whether a significant response was present in the subtracted PSTH, we used the method developed by Wiest et al. [47]. Briefly, the statistical significance of PSTHs was determined by comparing cumulative frequency histograms (CFHs) of PSTHs after sound onsets against the cumulative sum distribution of baseline PSTH before sound onsets ({−1, 0}s), estimated based on 1,000 bootstrapped samples (with replacement). The response onset latency was defined as the first bin in which postcue CFH exceeded the cumulative sum distribution from the baseline PSTH (*p* = 0.01, two-sided). A minimum response amplitude of 0.2 spike (per response) was required to be considered a significant response.

Fifty-nine percent (181/309) of recorded BF neurons showed a significant response within 200 ms of sound onset (Figure S3). Based on our previous studies [16],[21], we focused on BF neurons with a short latency (40–200 ms) excitatory response, which accounted for 162 of the 181 neurons. Furthermore, since the mean firing rate of the 162 neurons were bimodally distributed (Figure S3) and BF neurons encoding motivational salience have firing rates ≤8 spikes/s [16],[21], we included only the 144/162 neurons with firing rate ≤8 spikes/s as BF bursting neurons encoding motivational salience in our analysis. These neurons represented the most prominent neuronal response type in the BF.

Bursting amplitude was calculated as the mean firing rate within the {50,160} ms window after sound onset. There was considerable variability in the bursting amplitude across BF neurons (Figure 3C), even within the same recording session. In order to address the sampling variability and compare across sessions, we used the bursting amplitude ratio between S-Large and S-Small trials, which measures the modulation of BF bursting strength between the two conditions. By comparing this bursting modulation against RT modulation, we quantified how neuronal response modulations correlated with RT modulations between S-Large and S-Small trials. The activity of the entire bursting population (Figure 3D, Figure 4C and D) was calculated by pooling the activity of all BF bursting neurons recorded in a session as a multiunit.

Consistent with a decreasing baseline activity before sound onset, we noted that longer foreperiods produced a stronger activity decrease during the prestimulus period, accompanied by faster RTs (Figure S5). Thus, in our analysis comparing faster and slower trials within the same trial type (Figure 4), it was important to properly control for the influence of foreperiod on neuronal activity and on RT. We therefore first sorted trials associated with each foreperiod and then median split the trials into faster and slower halves (Figure S5). This procedure led to a proper matching of the foreperiod activity between the faster and slower half of trials.

### Electrical Stimulation

The behavior protocol (Figure 6A) for electrical stimulation was the same as the reward-biased simple RT task (Figure 1A) except that both S-Large and S-Small sounds were replaced by the same 6 kHz 80 dB tone in both trial types, but followed with or without a train of electrical stimulation delivered directly through the BF electrodes. Water reward, on both trial types, was the same (three drops of water). This design allowed us to assess whether the addition of BF electrical stimulation—as a way of augmenting the naturally occurring bursting response to the tone—could lead to faster RT distributions compared to nonstimulated control trials.

The stimulation was delivered through all 32 electrodes in the BF, the same electrode configuration as used in the recording experiment. This was intended to mimic the widespread presence of BF bursting neurons throughout the recording region, representing an ensemble bursting event of the entire population [16],[21].

Individual stimulation pulse was a biphasic charge-balanced pulse (0.1 ms each phase) delivered through a constant current stimulator (stimulus isolator A365R, World Precision Instruments, FL), driven by a Master-8-VP stimulator (A.M.P.I., Israel). Each stimulation train consisted of 11 pulses delivered at 100 Hz (10 ms interstimulus interval) and lasted a total of 100 ms. Stimulation current level was set at 16 µA, 24 µA, 32 µA, or 48 µA per electrode, resulting in a total of 0.5 mA to 1.5 mA over all electrodes. The timing of the stimulation was given at either {60,160} or {80,180} ms posttone onset to coincide with the BF bursting peak. We also implemented two stimulation current paths; one was a unipolar stimulation protocol with currents flowing between all BF electrodes (bilateral BF) against the reference skull screw over the cerebellum. In the second stimulation current path configuration, currents were flowing through all BF electrodes in one hemisphere against all BF electrodes in the other hemisphere.

In total, 44 sessions were tested in seven rats and 15 configurations, with 2–3 current levels tested in each configuration. One rat was first used for recording experiments in the reward-biased simple RT task, while the other six rats were never trained in the reward-biased simple RT task prior to the stimulation protocol. In Figure 6D, each gray line represents data collected from one rat, under one specific combination of current path and timing window. Linear mixed models were used to handle an unequal number of within-configuration observations in order to determine the influence of microstimulation on RT and RT modulation between stimulated and nonstimulated trials. The electrical stimulation current level was modeled as a continuous variable and its influence on RT modeled as a constant slope fixed effect. The choice of either timing windows and the choice of either current path configurations did not modulate RT differently (linear mixed models estimating the fixed effect of timing window on RT modulation, F(1,39) = 0.899, *p* = 0.349; and the fixed effect of current paths on RT modulation, F(1,39) = 0.303, *p* = 0.585; interaction term F(1,39) = 0.498, *p* = 0.485). Therefore, these variables were not included in the final model, which only tested whether the fixed effect of stimulation current level on RT was significantly different from zero. Electrical stimulation significantly decreased RT in stimulated trials as a function of stimulation current level (F(1,42) = 17.856, *p*<0.001; Figure 6C), but had no influence on RT in nonstimulated trials (F(1,42) = 0.235, *p* = 0.630; Figure 6C). Stimulation also significantly increased RT modulation (ratio of mean RT between nonstimulated and stimulated trials) as a function of stimulation current level (F(1,42) = 18.922, *p*<0.001; Figure 6D).

## Supporting Information

Figure S1 **Detailed behavioral characterization of RT differences between S-Large and S-Small trials in the reward-biased simple RT task.** (A–B) The entire response trajectory between sound onset to reward delivery was partitioned into four epochs. The modulation of response latencies between S-Large and S-Small trials in these four epochs were calculated as a ratio (A) or their difference (B) (mean ± sem, *n* = 16 rats, 339 sessions). The largest modulation was found in the earliest epoch corresponding to RT—that is, the latency between sound onset and fixation port exit. Repeated measure ANOVA and post hoc pair-wise comparisons showed significant differences in the mean between all pairs (*p*<0.001). (C) The average RT in S-Large and S-Small trials for each rat, averaged across all sessions per rat. Most rats have faster RTs in S-Large trials. (D) Boxplot of per session mean RT in S-Large trials for each rat. There exists a significant intersubject variability in decision speed. Rats are ranked by their mean RT in S-Large trials in both (C) and (D). (E–F) The number of rats (E) and the average number of sessions completed (F) at each reversal learning phase. Of the 16 total rats that acquired the task, 11/16 received first reversal learning and completed 19.1±11.6 (mean ± std) sessions of training (per rat) during first reversal learning. Five of 11 rats continued onto second reversal learning and completed 17.6±20.4 sessions. In total, 16 rats completed 25 reversal training transitions. The maximum number of contingency reversal was five (in two rats). (G–H) Mean RT for S-Small (G) and S-Large (H) trials relative to reversal learning transition. Convention as in Figure 1C. The red dotted lines indicate the overall average RT for S-Large trials across sessions. The mean RT for S-Large trials remains relatively stable throughout all phases of reversal learning, while the mean RT for S-Small trials shows significant modulation by reversal learning.
(TIF)

Figure S2 **Histological reconstruction of BF recording electrode locations.** (A) For each animal, the Nissl stain shows the most ventral location of the electrode bundle, indicated by the arrow. The reconstructed location of the electrode bundle is indicated by the red box. Because all 16 electrodes in one electrode bundle were moved together by the same microdrive, the location of individual recording electrode cannot be reconstructed. The spatial spread of electrodes at a particular depth was conservatively estimated to span no more than 1 mm (AP)×1 mm (ML)×0.5 mm (DV). Therefore, the box in each histological reconstruction represents the estimated spatial spread of electrodes throughout the entire dorso-ventral recording depth. The box is 1 mm wide (ML), and 0.25 mm was added to the most dorsal and most ventral recording depth to reflect uncertainty in the DV axis. Only one hemisphere is shown here for clarity. The zoom-in view shows the dorso-ventral extent of the recording depth, with each horizontal gray bar representing the estimated center location for one recording session. BF bursting neurons were recorded in 35/40 sessions. The locations of the five sessions in which no BF bursting neurons were recorded are indicated by black horizontal bars. (B) Summary of the histological reconstruction in the current study, with each color box representing one rat. The reconstruction shows that most BF bursting neurons were recorded from Rats 2–6, centered at Bregma −0.36 to −0.72 mm, throughout multiple subregions including the ventral part of GP, VP, SI, NBM, or B, MCPO, and HDB, but not in the adjacent hypothalamus region (LPO).
(TIF)

Figure S3 **Identification of BF bursting neurons.** (A) PSTHs of all BF neurons (*n* = 309) aligned to sound onset (left) and PSTHs in catch trials aligned to matching foreperiods when the sound onset would have occurred (right). BF neurons were sorted by their response onset latency, starting with excitatory responses and followed by inhibitory responses. (B) PSTHs of all BF neurons with the responses in catch trials subtracted. The red bar to the right indicates the 162 neurons with short latency (<200 ms) excitatory response to sound onset. Nineteen BF neurons showed short latency (<200 ms) inhibitory response to sound onset. (C) The mean firing rate of the 162 neurons with short latency excitatory response plotted on log scale showed a bimodal distribution. The 144 neurons with mean firing rate <8 spikes/s were identified as BF bursting neurons and selected for further analysis.
(TIF)

Figure S4 **Correlation between BF bursting amplitude and absolute RT.** (A–C) Correlation between the population BF bursting amplitude and mean RT of S-Large trials (A), S-Small trials (B), and both trial types combined (C), in each session. Results plotted separately for individual BF bursting neurons (gray), as well as for the entire bursting population (red and green) per session. Linear regression was shown using the population BF bursting amplitude per session. Unlike the results in Figure 3D, there was very weak correlation between the BF bursting amplitude and the absolute mean RT. The weak correlations here likely reflect two factors: First, there exists a substantial variability in the bursting amplitude among salience-encoding BF neurons (Figure S3), and hence a significant sampling variability of BF activity across sessions. Second, there exists a significant intersubject variability in decision speed (Figure S1D), which is determined by factors other than the motivational salience of the sounds. The ratio measures we used in Figure 3D provided an internal normalization of these between-session variabilities and isolated the contribution of BF motivational salience signal in modulating decision speed.
(TIF)

Figure S5 **The effect of foreperiod on BF activity.** (A) The population PSTHs of BF bursting neurons were plotted for S-Large (top) and S-Small trials (bottom), calculated separately for each foreperiod. FP1 was the shortest 0.35 s foreperiod, whereas FP4 was the longest 0.80 s foreperiod. The mean RTs for the corresponding trials are indicated in the inset (mean ± std). Longer foreperiod was associated with faster RTs, but did not increase BF bursting amplitude, suggesting that faster RTs associated with longer foreperiods were not mediated by increased BF bursting amplitude. Instead, longer foreperiod was associated with stronger prestimulus activity reduction. (B) An example BF bursting neuron illustrating how faster and slower trials were determined for each foreperiod. This procedure controlled for the influence of foreperiod on BF activity and on RT.
(TIF)

Figure S6 **The difference in BF bursting amplitude between faster and slower trials within a trial type.** (A–C) The difference (mean ± sem) in BF bursting amplitude between faster and slower RTs within S-Large (A) and S-Small (B) trials, and between S-Large and S-Small trials (C) (paired *t* test). The average bursting amplitude difference was indicated. (D–F) The same BF bursting amplitude difference analysis as in (A–C), except that the respective baseline firing rate at {−100,0} ms window was first subtracted. Adjusting for the baseline firing rate resulted in smaller and less significant BF bursting amplitude difference, indicating that the small difference in BF bursting amplitude between faster and slower trials within a trial type was partly contributed by the difference in baseline activity.
(TIF)

Figure S7 **Schematic of recinormal RT distribution and K-S fit.** Although a recinormal RT distribution (A) is skewed to the right, the reciprocal of which (1/RT) is normally distributed, with mean μ and standard deviation σ (B). Note that the *x*-axis in (B) is reversed so that faster trials were plotted to the left of the distribution. Plotting (−1/RT) against its *z*-score (C) transforms the RT distribution into a straight line, expressed by the equation y = (x+μ)/σ. The LATER model is depicted in the bottom half of (A), indicating that RTs can be generated by a stochastic neural process that accumulates activity at a constant rise rate until reaching a decision threshold. The rise rate is randomly drawn from the normal distribution (1/RT) in each trial. K-S test was used to compare the empirical RT distribution with the fitted recinormal RT distribution, and to determine the best estimate of the μ and σ parameters that produced the minimal *p* values for the K-S test. Scatter plot of the K-S test *p* values for the two RT distributions in a session (D) shows that most RT distributions were well described by recinormal distributions. A total of 339/431 sessions had both *p* values≥0.6 (red dashed lines) and were selected for final behavioral analysis. Each dot represents data in one session from one rat. (E) RT distributions in four example sessions from one rat sampled at different sessions around reversal learning transition are shown to better understand the relationship between S-Large and S-Small recinormal RT distributions throughout the course of reversal learning. In the last session before reversal (left panel), the large RT modulation between two trial types was reflected by the large separation between the two RT distributions. After reversal learning transition (2nd–4th panels), the separation between the two RT distributions decreased and slowly reemerged after several sessions.
(TIF)

Figure S8 **Histological reconstruction of the locations of BF stimulation electrodes. Convention as in Figure S2.** Rats S1–S6 were used exclusively for BF electrical stimulation experiment, and Rat 6 was used initially for BF recording in the reward-biased simple RT task. The reconstructed locations for all rats are overlaid on the same sections in the bottom summary panel, with each color representing one rat. The locations of BF electrical stimulation electrodes collectively cover similar regions as the BF recording electrodes (Figure S2), which is consistent with the location of cortically projecting BF neurons as revealed by placing retrograde tracers in the prefrontal cortex [28].
(TIF)

Figure S9 **BF electrical stimulation preserves the coupling between μ and σ parameters of RT distributions.** (A) Our model in Figure 5F predicts that manipulating the amplitude of BF bursting should modulate both the speed and variability parameters of the RT distribution while the intersection point should remain unchanged. To further investigate whether BF electrical stimulation shifted the coupling between μ and σ parameters of RT distributions and the estimated intersection point shown in Figure 5D, we first reproduced Figure 5D here for comparison. Each dot is derived from RT distributions in one session of reward-biased simple RT task (*n* = 339, 16 rats). The invariant intersection point is estimated to be −1/−6.33 = 158 ms (−1/slope). (B) The same intersection point analysis was applied to all 44 BF electrical stimulation sessions, using the same method to estimate μ and σ parameters (see the dotted green line in panel C for an example). The blue line represents the significant linear regression, which had a much lower slope (−2.25) compared to the linear regression slope in (A), shown here as the black dotted line for comparison. We note that, however, most sessions are well described by the original linear regression (black dotted line). (C) Another example session shows the influence of BF stimulation on the RT distribution. Convention as in Figure 6B. Closer examination of RT distributions in BF stimulation sessions found that although RTs in stimulated trials were faster compared to tone alone trials (Figure 6C–D), RTs in stimulated trials slowed down significantly after ∼250 ms after tone onset (or 70–90 ms after the end of BF electrical stimulation) like the example session shown here. RTs longer than 250 ms were much slower than expected based on the recinormal RT distribution constructed by RTs faster than 250 ms. The significant slowing of long latency RTs in BF-stimulated trials likely resulted from an unintended consequence of BF electrical stimulation, which induced a delayed-onset near-complete inhibition of salience-encoding BF neurons starting at 60–80 ms after BF electrical stimulation (unpublished data). To eliminate the confounding influence of delayed inhibition on RTs and to appropriately assess how enhancing BF bursting amplitude via BF electrical stimulation modulates RT, we modified our estimation of the μ and σ parameters of RT distributions based only on RTs faster than 250 ms (solid green line). (D) Using this revised method, we re-estimated μ and σ parameters in 32/44 sessions that included at least 10 stimulated trials with RT faster than 250 ms. Most of the outlier sessions in (B) did not have enough trials and were removed from the analysis, which suggests that RTs in those sessions are likely driven by the unintended delayed inhibition of BF neurons. Using the revised estimate of μ and σ parameters, we found a much stronger linear regression and estimated the intersection point to be −1/−5.31 = 188 ms, comparable to what we found in (A).
(TIF)

Figure S10 **BF bursting response to trial start light signal.** (A) An example BF bursting neuron showing bursting responses to the trial start light signal. Trials were aligned to light offset and sorted based on the latency between light offset to fixation port entry (green). The response latency to the trial start signal provided a proxy for the initial RT, but was confounded by the variable starting position of the animal at the time of light offset. As commonly seen in BF bursting neurons, stronger BF bursting response was associated with shorter response latency on a trial-by-trial basis, which supports the idea that stronger BF bursting leads to faster decision speed and shorter RT. Unlike the similar BF bursting amplitude between faster and slower trials within S-Large or S-Small trials, BF bursting amplitude showed large fluctuation across trials because rats were not required to maintain fixation and their behavioral states at the time of light offset were not constrained. Therefore, the fluctuation in BF bursting amplitude likely reflected the influence of fluctuations in arousal, fatigue, or satiety on motivational salience. (B) Population PSTH to the trial start light signal (mean ± sem, *n* = 144) in trials with faster and slower response. BF bursting amplitude was larger in trials with shorter response latency. Furthermore, faster responses to the trial start light signal were associated with lower prestimulus baseline firing rate at {−300, 0} ms before the light signal. This pattern is similar to the observation that longer foreperiods were associated with faster RTs and stronger prestimulus activity reduction (Figure S5), suggesting the possibility that lower prestimulus activity of salience-encoding BF neurons may be associated with faster RTs, and the reduction of prestimulus activity may be modulated by a temporal expectation signal. The significant difference in prestimulus activity also supports the idea that the behavioral state of the animal at the time of light offset was different between faster and slower trials to the trial start light signal.
(TIF)

## Abbreviations

BF: basal forebrain
GP: globus pallidus
HDB: horizontal diagonal band
IR: infrared
ITI: intertrial interval
K-S: Kolmogorov-Smirnov test
LPO: lateral preoptic area
MCPO: magnocellular preoptic nucleus
NBM or B: nucleus basalis of Meynert
PSTH: peri-stimulus time histogram
RT: reaction time
SI: substantia innominata
S-Large: sound predicting a large reward
S-Small: sound predicting a small reward
VP: ventral pallidum
μ: speed parameter of recinormal RT distribution
σ: variability parameter of recinormal RT distribution
