# Supplementary figures and images for: Motivational Salience Signal in the Basal Forebrain Is Coupled with Faster and More Precise Decision Speed
Source: PLoS Biol. 2014 Mar 18;12(3):e1001811. doi: 10.1371/journal.pbio.1001811 (PMC3958335; doi:10.1371/journal.pbio.1001811)

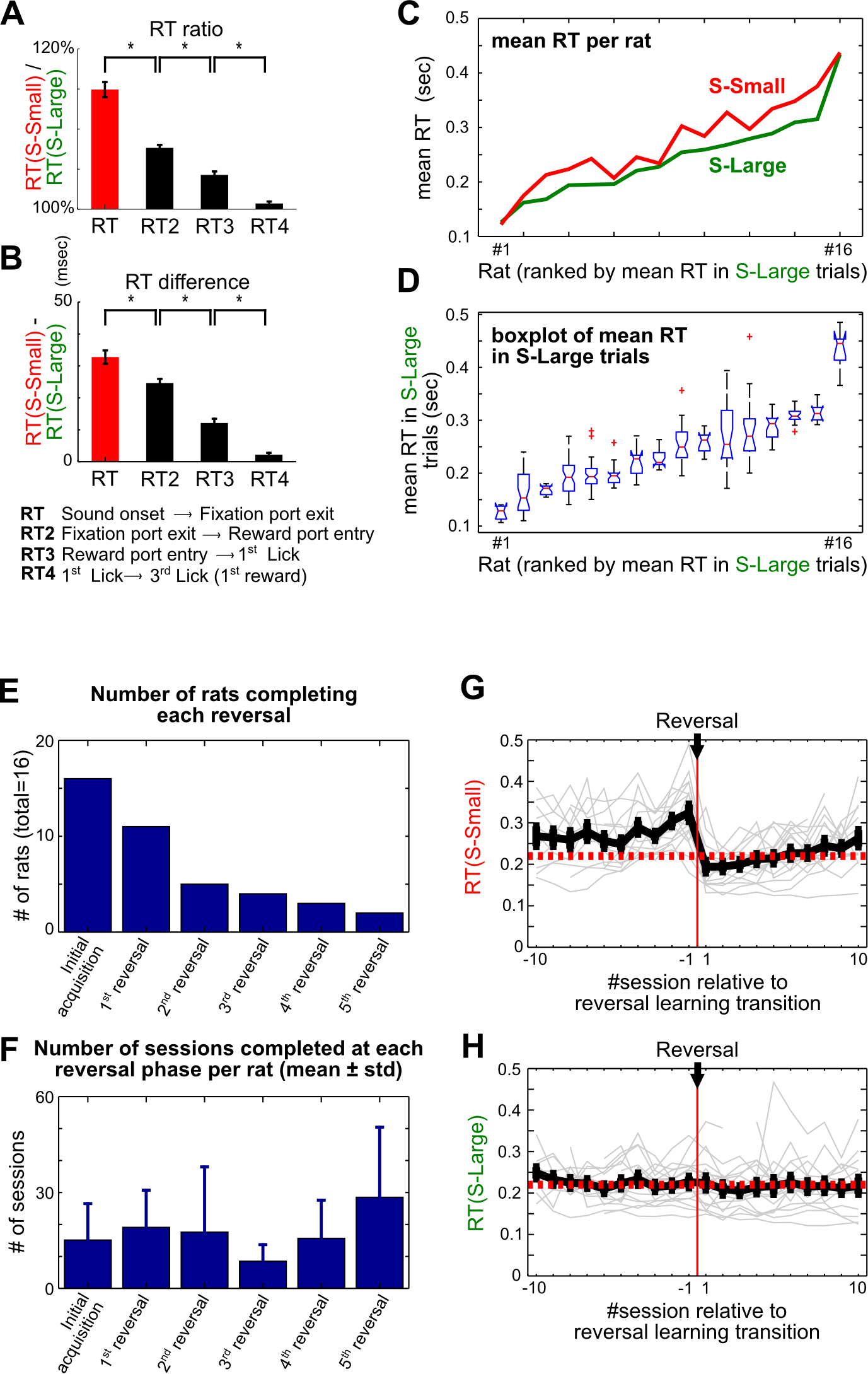

Supplement: Figure S1 — Detailed behavioral characterization of RT differences between S-Large and S-Small trials in the reward-biased simple RT task. (A–B) The entire response trajectory between sound onset to reward delivery was partitioned into four epochs. The modulation of response latencies between S-Large and S-Small trials in these four epochs were calculated as a ratio (A) or their difference (B) (mean ± sem, n = 16 rats, 339 sessions). The largest modulation was found in the earliest epoch corresponding to RT—that is, the latency between sound onset and fixation port exit. Repeated measure ANOVA and post hoc pair-wise comparisons showed significant differences in the mean between all pairs (p<0.001). (C) The average RT in S-Large and S-Small trials for each rat, averaged across all sessions per rat. Most rats have faster RTs in S-Large trials. (D) Boxplot of per session mean RT in S-Large trials for each rat. There exists a significant intersubject variability in decision speed. Rats are ranked by their mean RT in S-Large trials in both (C) and (D). (E–F) The number of rats (E) and the average number of sessions completed (F) at each reversal learning phase. Of the 16 total rats that acquired the task, 11/16 received first reversal learning and completed 19.1±11.6 (mean ± std) sessions of training (per rat) during first reversal learning. Five of 11 rats continued onto second reversal learning and completed 17.6±20.4 sessions. In total, 16 rats completed 25 reversal training transitions. The maximum number of contingency reversal was five (in two rats). (G–H) Mean RT for S-Small (G) and S-Large (H) trials relative to reversal learning transition. Convention as in Figure 1C. The red dotted lines indicate the overall average RT for S-Large trials across sessions. The mean RT for S-Large trials remains relatively stable throughout all phases of reversal learning, while the mean RT for S-Small trials shows significant modulation by reversal learning. (TIF) [file pbio.1001811.s001.tif]

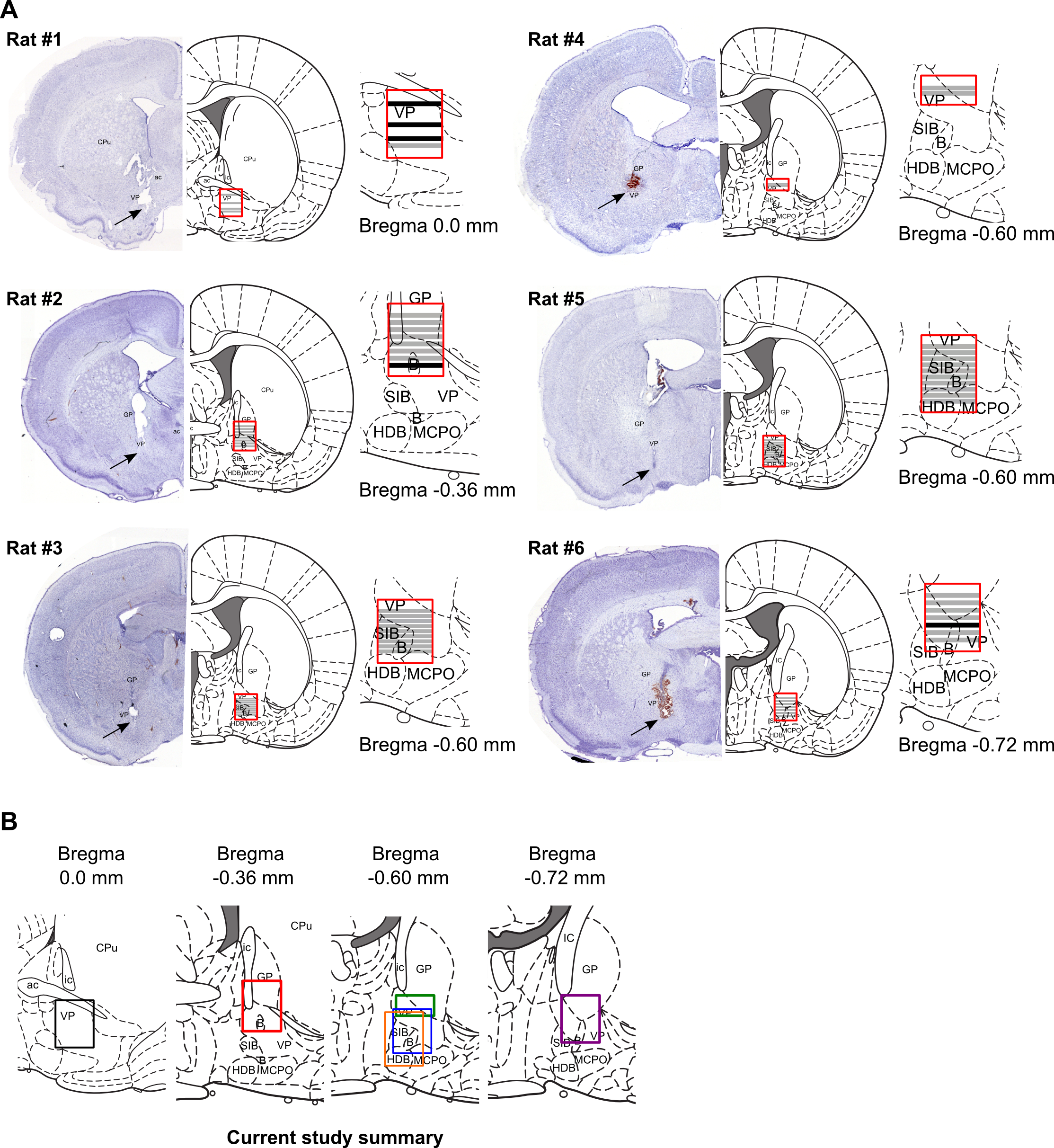

Supplement: Figure S2 — Histological reconstruction of BF recording electrode locations. (A) For each animal, the Nissl stain shows the most ventral location of the electrode bundle, indicated by the arrow. The reconstructed location of the electrode bundle is indicated by the red box. Because all 16 electrodes in one electrode bundle were moved together by the same microdrive, the location of individual recording electrode cannot be reconstructed. The spatial spread of electrodes at a particular depth was conservatively estimated to span no more than 1 mm (AP)×1 mm (ML)×0.5 mm (DV). Therefore, the box in each histological reconstruction represents the estimated spatial spread of electrodes throughout the entire dorso-ventral recording depth. The box is 1 mm wide (ML), and 0.25 mm was added to the most dorsal and most ventral recording depth to reflect uncertainty in the DV axis. Only one hemisphere is shown here for clarity. The zoom-in view shows the dorso-ventral extent of the recording depth, with each horizontal gray bar representing the estimated center location for one recording session. BF bursting neurons were recorded in 35/40 sessions. The locations of the five sessions in which no BF bursting neurons were recorded are indicated by black horizontal bars. (B) Summary of the histological reconstruction in the current study, with each color box representing one rat. The reconstruction shows that most BF bursting neurons were recorded from Rats 2–6, centered at Bregma −0.36 to −0.72 mm, throughout multiple subregions including the ventral part of GP, VP, SI, NBM, or B, MCPO, and HDB, but not in the adjacent hypothalamus region (LPO). (TIF) [file pbio.1001811.s002.tif]

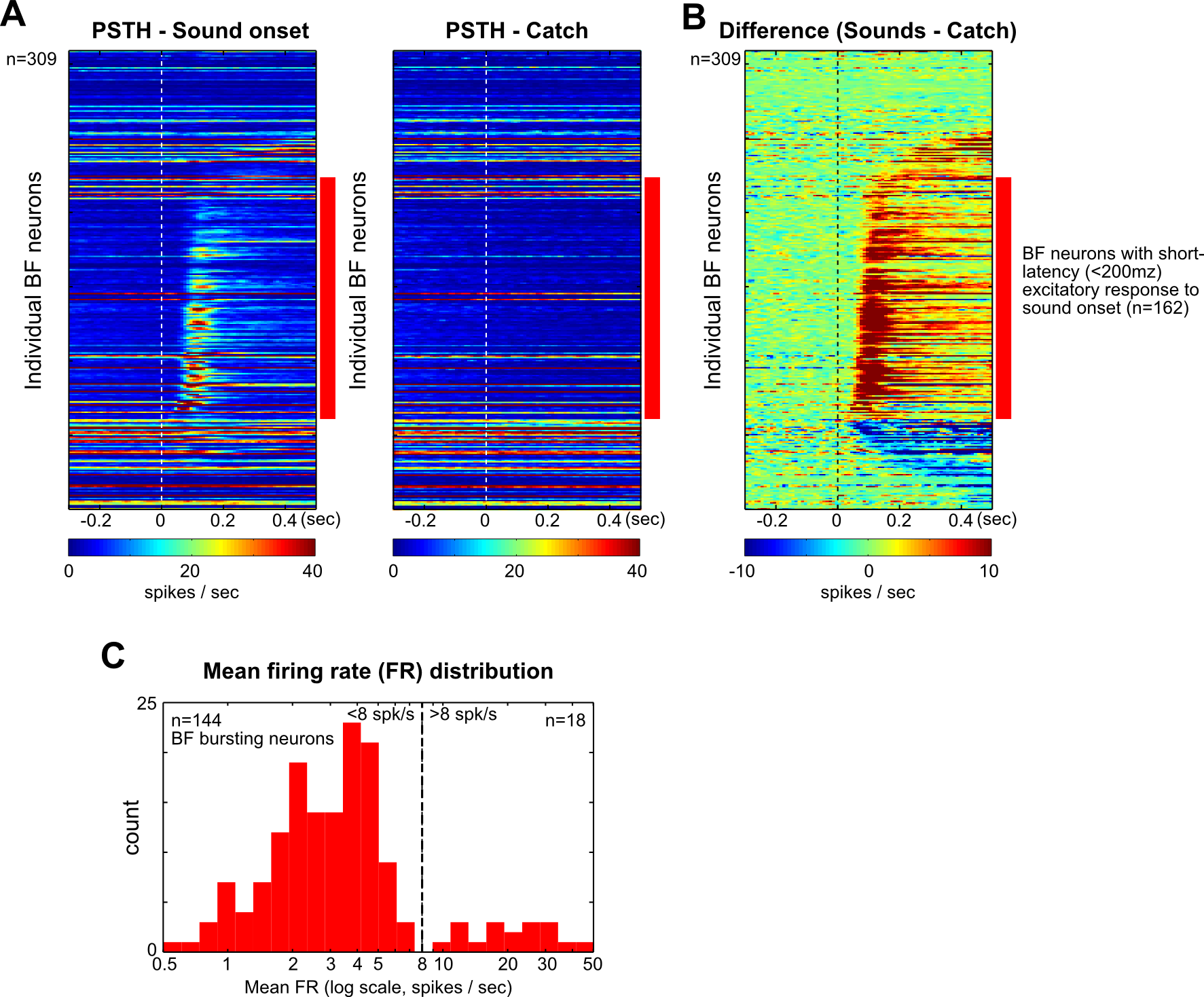

Supplement: Figure S3 — Identification of BF bursting neurons. (A) PSTHs of all BF neurons (n = 309) aligned to sound onset (left) and PSTHs in catch trials aligned to matching foreperiods when the sound onset would have occurred (right). BF neurons were sorted by their response onset latency, starting with excitatory responses and followed by inhibitory responses. (B) PSTHs of all BF neurons with the responses in catch trials subtracted. The red bar to the right indicates the 162 neurons with short latency (<200 ms) excitatory response to sound onset. Nineteen BF neurons showed short latency (<200 ms) inhibitory response to sound onset. (C) The mean firing rate of the 162 neurons with short latency excitatory response plotted on log scale showed a bimodal distribution. The 144 neurons with mean firing rate <8 spikes/s were identified as BF bursting neurons and selected for further analysis. (TIF) [file pbio.1001811.s003.tif]

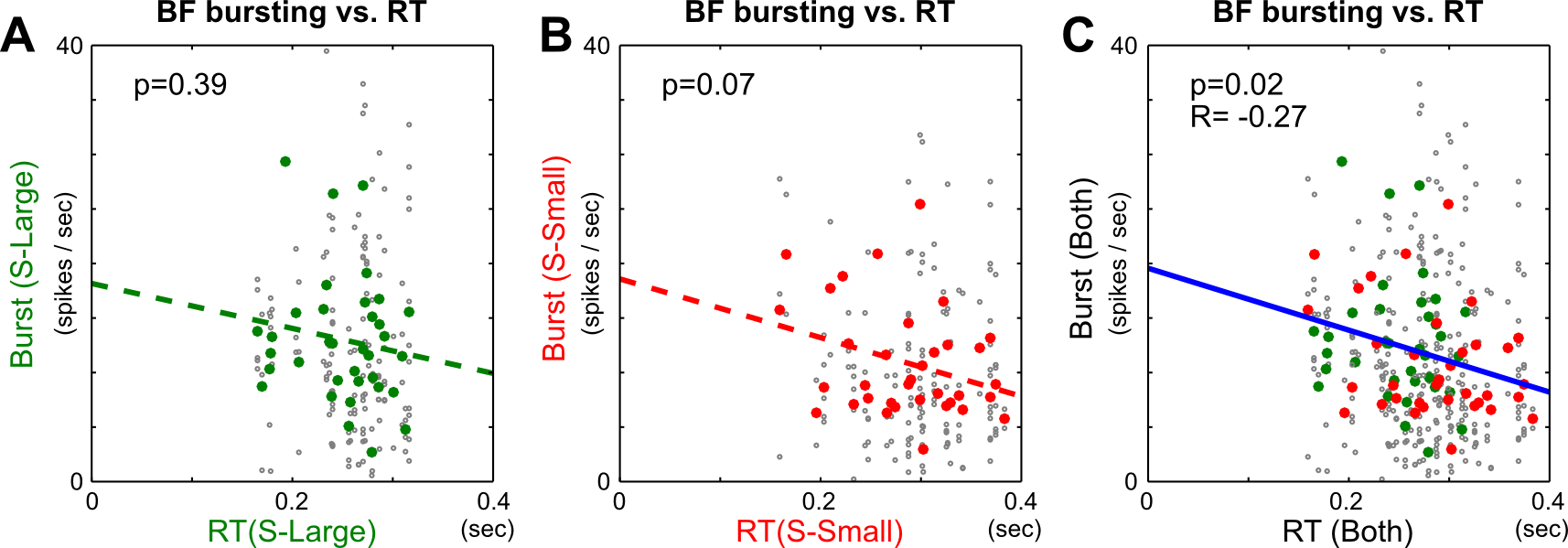

Supplement: Figure S4 — Correlation between BF bursting amplitude and absolute RT. (A–C) Correlation between the population BF bursting amplitude and mean RT of S-Large trials (A), S-Small trials (B), and both trial types combined (C), in each session. Results plotted separately for individual BF bursting neurons (gray), as well as for the entire bursting population (red and green) per session. Linear regression was shown using the population BF bursting amplitude per session. Unlike the results in Figure 3D, there was very weak correlation between the BF bursting amplitude and the absolute mean RT. The weak correlations here likely reflect two factors: First, there exists a substantial variability in the bursting amplitude among salience-encoding BF neurons (Figure S3), and hence a significant sampling variability of BF activity across sessions. Second, there exists a significant intersubject variability in decision speed (Figure S1D), which is determined by factors other than the motivational salience of the sounds. The ratio measures we used in Figure 3D provided an internal normalization of these between-session variabilities and isolated the contribution of BF motivational salience signal in modulating decision speed. (TIF) [file pbio.1001811.s004.tif]

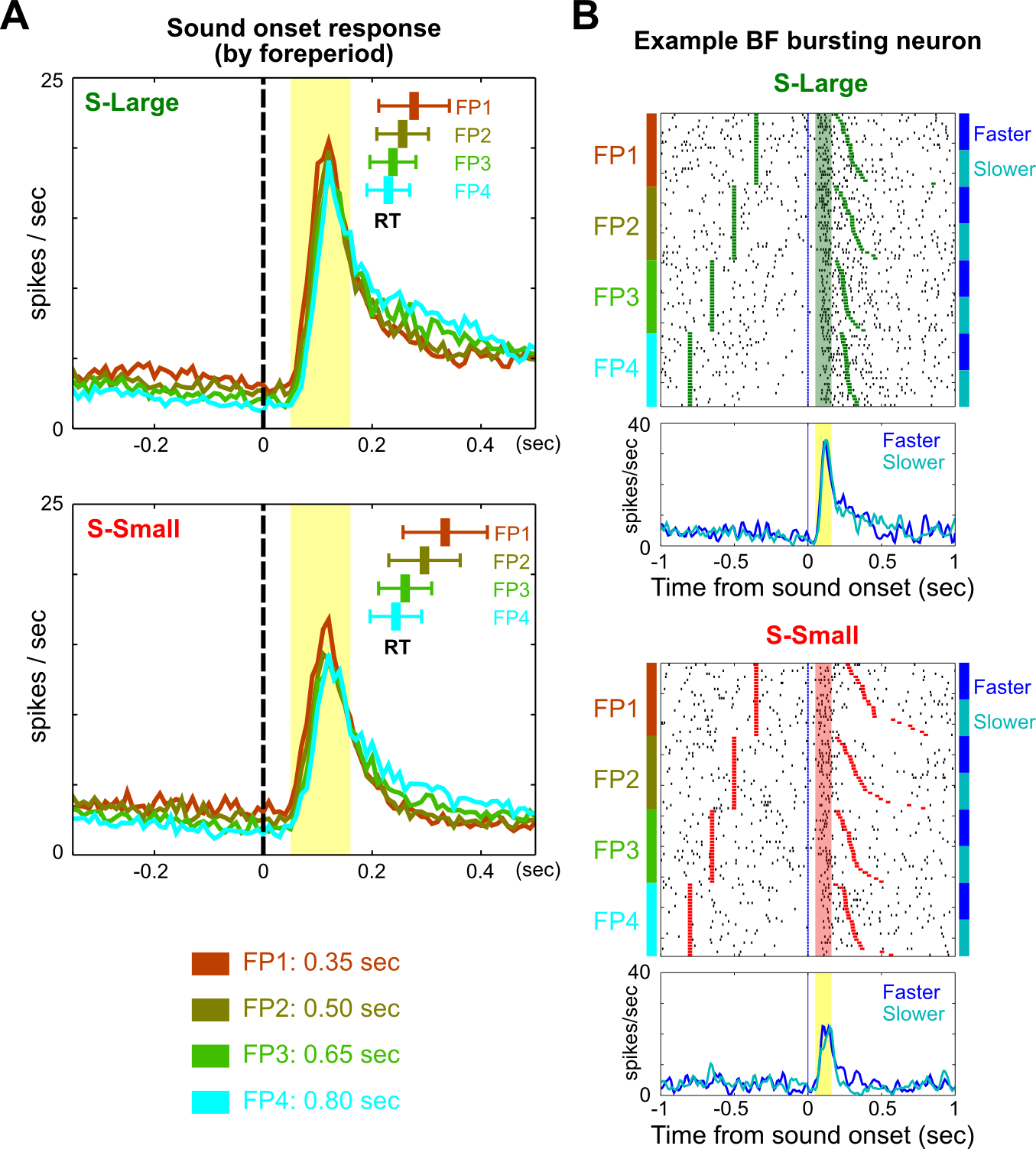

Supplement: Figure S5 — The effect of foreperiod on BF activity. (A) The population PSTHs of BF bursting neurons were plotted for S-Large (top) and S-Small trials (bottom), calculated separately for each foreperiod. FP1 was the shortest 0.35 s foreperiod, whereas FP4 was the longest 0.80 s foreperiod. The mean RTs for the corresponding trials are indicated in the inset (mean ± std). Longer foreperiod was associated with faster RTs, but did not increase BF bursting amplitude, suggesting that faster RTs associated with longer foreperiods were not mediated by increased BF bursting amplitude. Instead, longer foreperiod was associated with stronger prestimulus activity reduction. (B) An example BF bursting neuron illustrating how faster and slower trials were determined for each foreperiod. This procedure controlled for the influence of foreperiod on BF activity and on RT. (TIF) [file pbio.1001811.s005.tif]

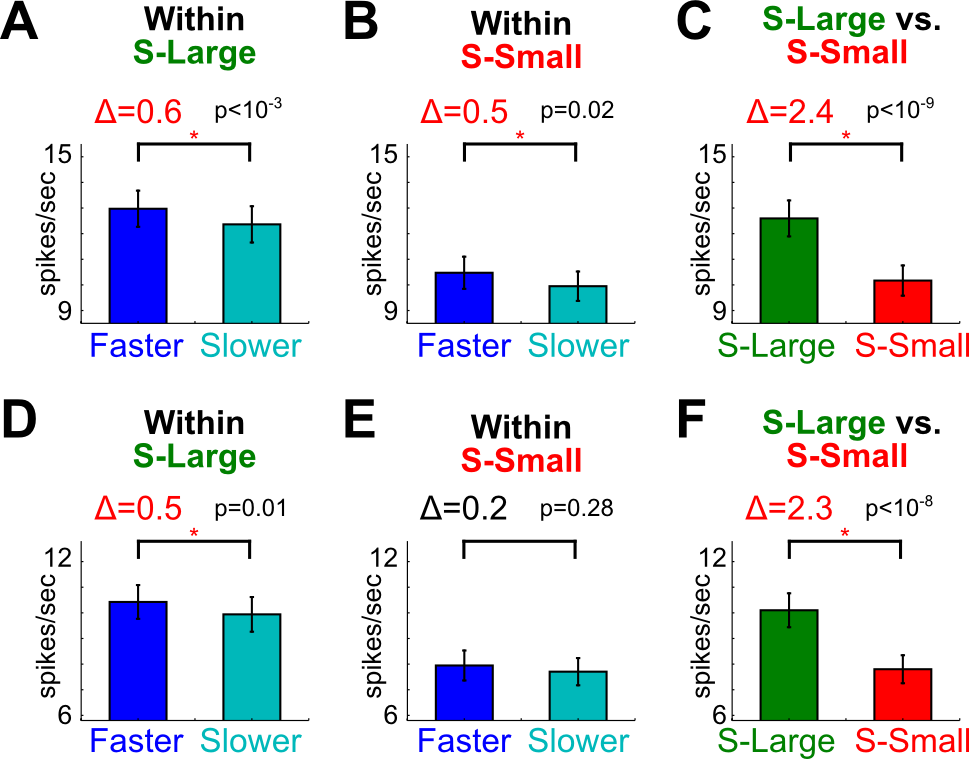

Supplement: Figure S6 — The difference in BF bursting amplitude between faster and slower trials within a trial type. (A–C) The difference (mean ± sem) in BF bursting amplitude between faster and slower RTs within S-Large (A) and S-Small (B) trials, and between S-Large and S-Small trials (C) (paired t test). The average bursting amplitude difference was indicated. (D–F) The same BF bursting amplitude difference analysis as in (A–C), except that the respective baseline firing rate at {−100,0} ms window was first subtracted. Adjusting for the baseline firing rate resulted in smaller and less significant BF bursting amplitude difference, indicating that the small difference in BF bursting amplitude between faster and slower trials within a trial type was partly contributed by the difference in baseline activity. (TIF) [file pbio.1001811.s006.tif]

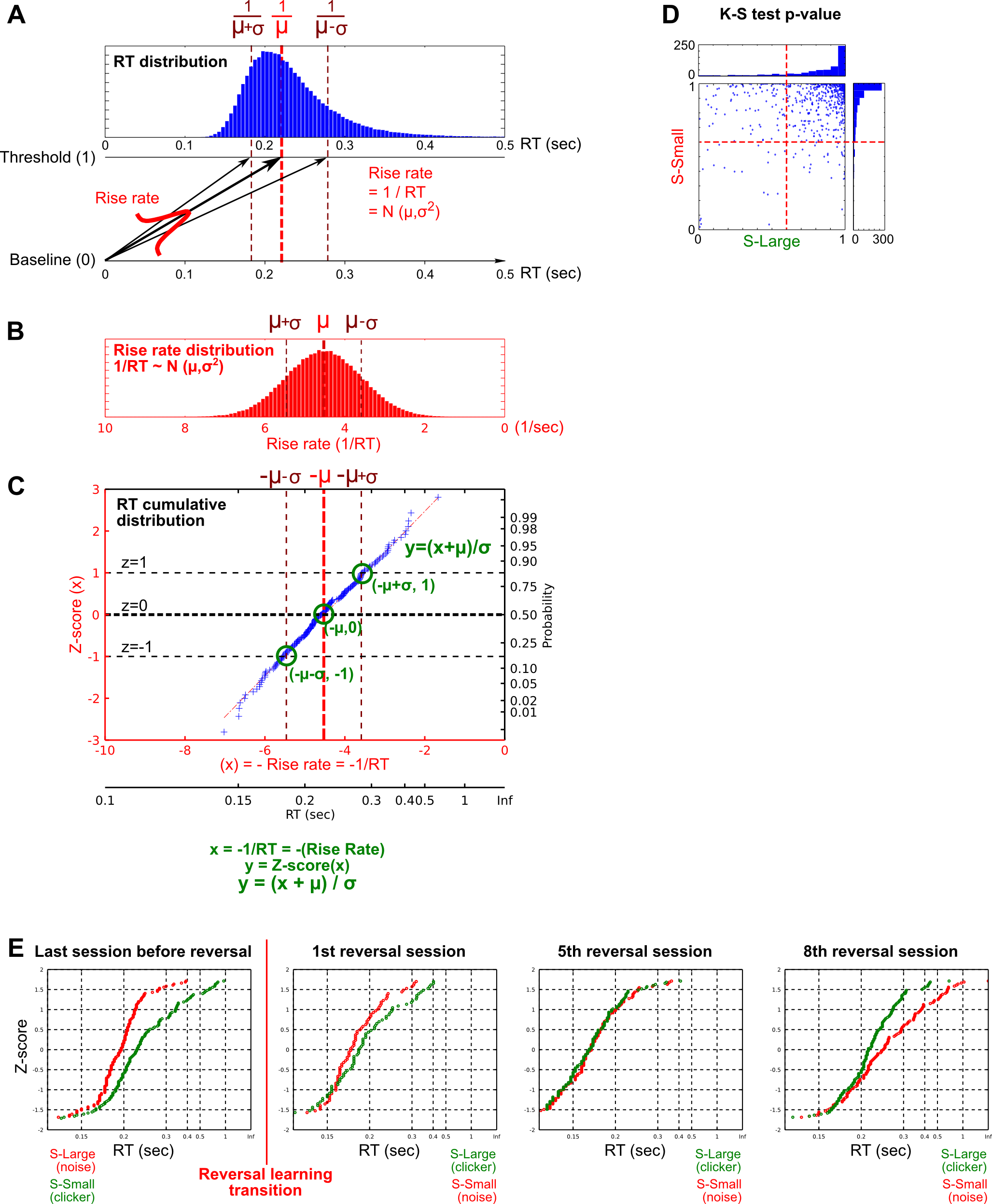

Supplement: Figure S7 — Schematic of recinormal RT distribution and K-S fit. Although a recinormal RT distribution (A) is skewed to the right, the reciprocal of which (1/RT) is normally distributed, with mean μ and standard deviation σ (B). Note that the x-axis in (B) is reversed so that faster trials were plotted to the left of the distribution. Plotting (−1/RT) against its z-score (C) transforms the RT distribution into a straight line, expressed by the equation y = (x+μ)/σ. The LATER model is depicted in the bottom half of (A), indicating that RTs can be generated by a stochastic neural process that accumulates activity at a constant rise rate until reaching a decision threshold. The rise rate is randomly drawn from the normal distribution (1/RT) in each trial. K-S test was used to compare the empirical RT distribution with the fitted recinormal RT distribution, and to determine the best estimate of the μ and σ parameters that produced the minimal p values for the K-S test. Scatter plot of the K-S test p values for the two RT distributions in a session (D) shows that most RT distributions were well described by recinormal distributions. A total of 339/431 sessions had both p values≥0.6 (red dashed lines) and were selected for final behavioral analysis. Each dot represents data in one session from one rat. (E) RT distributions in four example sessions from one rat sampled at different sessions around reversal learning transition are shown to better understand the relationship between S-Large and S-Small recinormal RT distributions throughout the course of reversal learning. In the last session before reversal (left panel), the large RT modulation between two trial types was reflected by the large separation between the two RT distributions. After reversal learning transition (2nd–4th panels), the separation between the two RT distributions decreased and slowly reemerged after several sessions. (TIF) [file pbio.1001811.s007.tif]

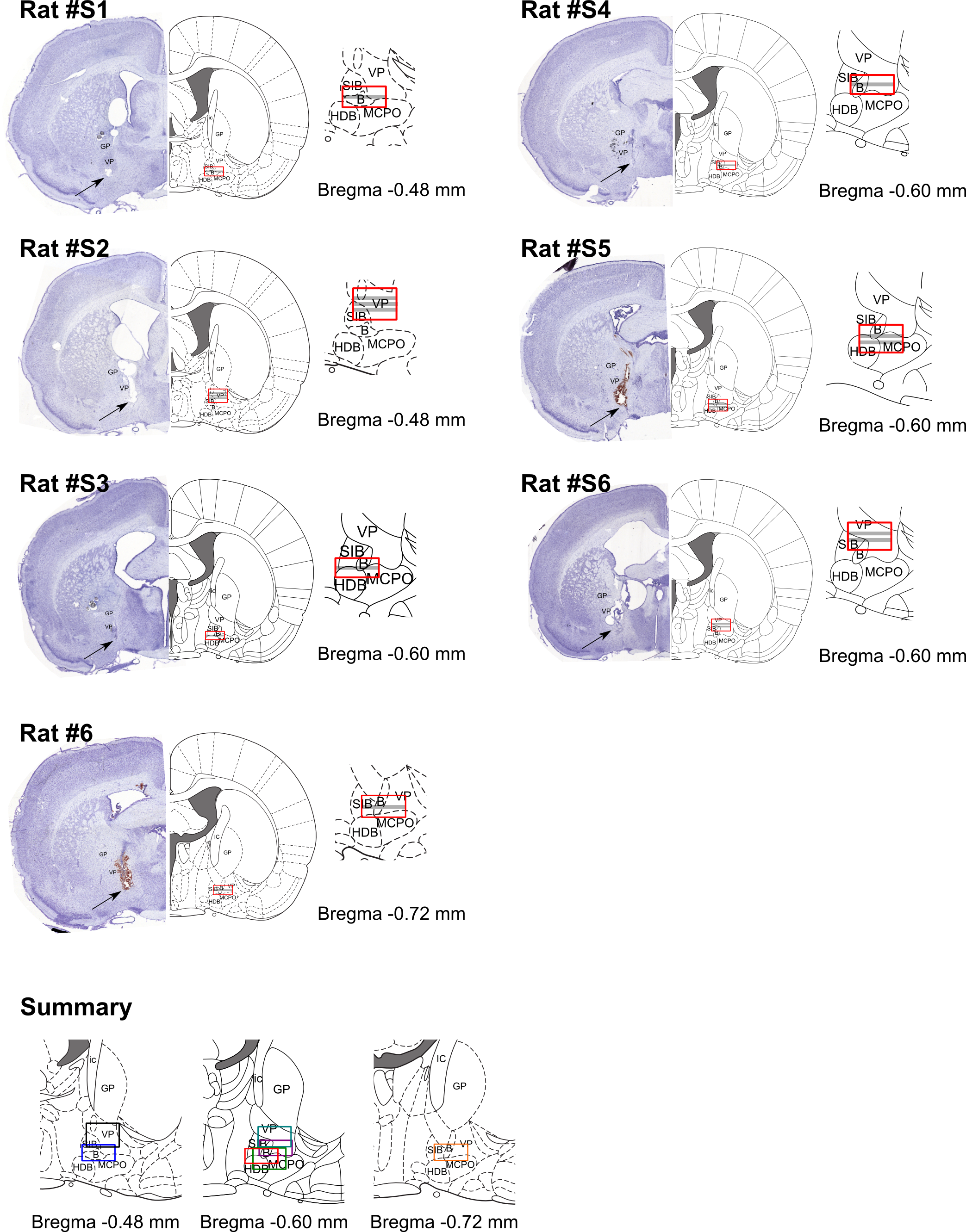

Supplement: Figure S8 — Histological reconstruction of the locations of BF stimulation electrodes. Convention as in Figure S2. Rats S1–S6 were used exclusively for BF electrical stimulation experiment, and Rat 6 was used initially for BF recording in the reward-biased simple RT task. The reconstructed locations for all rats are overlaid on the same sections in the bottom summary panel, with each color representing one rat. The locations of BF electrical stimulation electrodes collectively cover similar regions as the BF recording electrodes (Figure S2), which is consistent with the location of cortically projecting BF neurons as revealed by placing retrograde tracers in the prefrontal cortex [28]. (TIF) [file pbio.1001811.s008.tif]

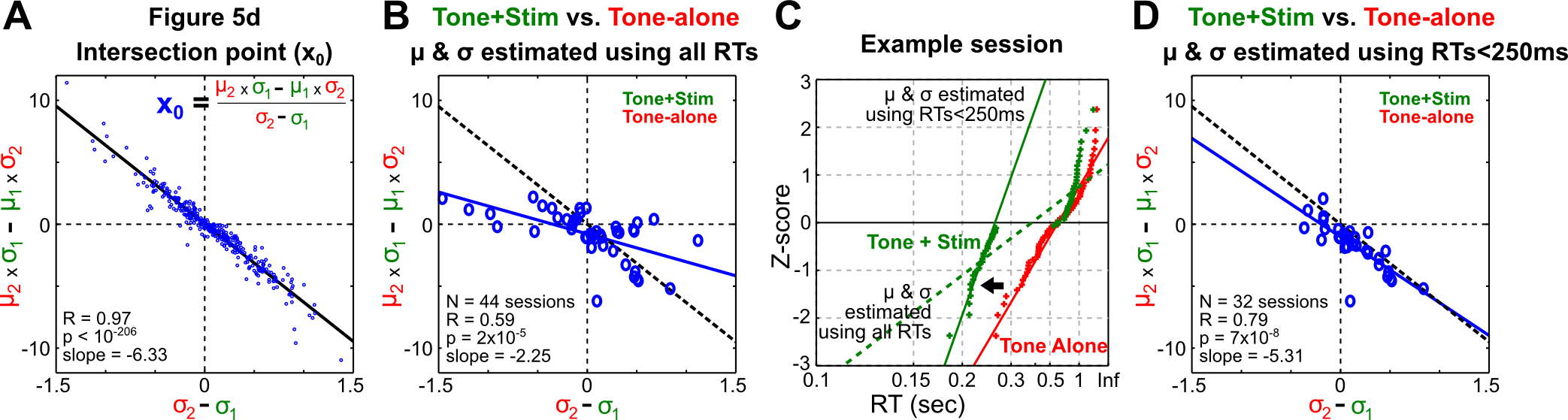

Supplement: Figure S9 — BF electrical stimulation preserves the coupling between μ and σ parameters of RT distributions. (A) Our model in Figure 5F predicts that manipulating the amplitude of BF bursting should modulate both the speed and variability parameters of the RT distribution while the intersection point should remain unchanged. To further investigate whether BF electrical stimulation shifted the coupling between μ and σ parameters of RT distributions and the estimated intersection point shown in Figure 5D, we first reproduced Figure 5D here for comparison. Each dot is derived from RT distributions in one session of reward-biased simple RT task (n = 339, 16 rats). The invariant intersection point is estimated to be −1/−6.33 = 158 ms (−1/slope). (B) The same intersection point analysis was applied to all 44 BF electrical stimulation sessions, using the same method to estimate μ and σ parameters (see the dotted green line in panel C for an example). The blue line represents the significant linear regression, which had a much lower slope (−2.25) compared to the linear regression slope in (A), shown here as the black dotted line for comparison. We note that, however, most sessions are well described by the original linear regression (black dotted line). (C) Another example session shows the influence of BF stimulation on the RT distribution. Convention as in Figure 6B. Closer examination of RT distributions in BF stimulation sessions found that although RTs in stimulated trials were faster compared to tone alone trials (Figure 6C–D), RTs in stimulated trials slowed down significantly after ∼250 ms after tone onset (or 70–90 ms after the end of BF electrical stimulation) like the example session shown here. RTs longer than 250 ms were much slower than expected based on the recinormal RT distribution constructed by RTs faster than 250 ms. The significant slowing of long latency RTs in BF-stimulated trials likely resulted from an unintended consequence of BF electrical stimulation, which [file pbio.1001811.s009.tif]

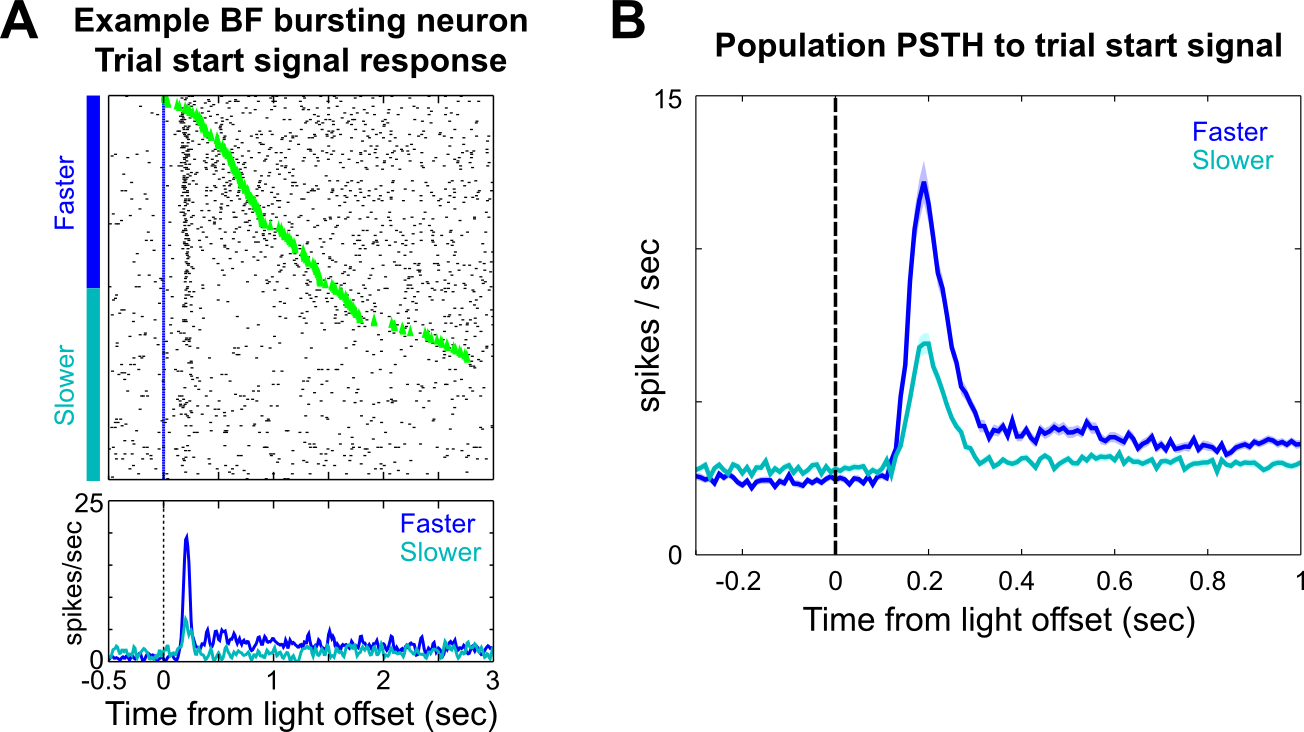

Supplement: Figure S10 — BF bursting response to trial start light signal. (A) An example BF bursting neuron showing bursting responses to the trial start light signal. Trials were aligned to light offset and sorted based on the latency between light offset to fixation port entry (green). The response latency to the trial start signal provided a proxy for the initial RT, but was confounded by the variable starting position of the animal at the time of light offset. As commonly seen in BF bursting neurons, stronger BF bursting response was associated with shorter response latency on a trial-by-trial basis, which supports the idea that stronger BF bursting leads to faster decision speed and shorter RT. Unlike the similar BF bursting amplitude between faster and slower trials within S-Large or S-Small trials, BF bursting amplitude showed large fluctuation across trials because rats were not required to maintain fixation and their behavioral states at the time of light offset were not constrained. Therefore, the fluctuation in BF bursting amplitude likely reflected the influence of fluctuations in arousal, fatigue, or satiety on motivational salience. (B) Population PSTH to the trial start light signal (mean ± sem, n = 144) in trials with faster and slower response. BF bursting amplitude was larger in trials with shorter response latency. Furthermore, faster responses to the trial start light signal were associated with lower prestimulus baseline firing rate at {−300, 0} ms before the light signal. This pattern is similar to the observation that longer foreperiods were associated with faster RTs and stronger prestimulus activity reduction (Figure S5), suggesting the possibility that lower prestimulus activity of salience-encoding BF neurons may be associated with faster RTs, and the reduction of prestimulus activity may be modulated by a temporal expectation signal. The significant difference in prestimulus activity also supports the idea that the behavioral state of the animal at the time of l [file pbio.1001811.s010.tif]
